# Real-Time Amperometric Recording of Extracellular H_2_O_2_ in the Brain of Immunocompromised Mice: An In Vitro, Ex Vivo and In Vivo Characterisation Study

**DOI:** 10.3390/s17071596

**Published:** 2017-07-08

**Authors:** Caroline H. Reid, Niall J. Finnerty

**Affiliations:** Chemistry Department, Maynooth University, Maynooth W23 F2H6, County Kildare, Ireland; caroline.reid@nuim.ie

**Keywords:** amperometry, H_2_O_2_, characterisation, immunocompromised, in vivo, extracellular

## Abstract

We detail an extensive characterisation study on a previously described dual amperometric H_2_O_2_ biosensor consisting of H_2_O_2_ detection (blank) and degradation (catalase) electrodes. In vitro investigations demonstrated excellent H_2_O_2_ sensitivity and selectivity against the interferent, ascorbic acid. Ex vivo studies were performed to mimic physiological conditions prior to in vivo deployment. Exposure to brain tissue homogenate identified reliable sensitivity and selectivity recordings up to seven days for both blank and catalase electrodes. Furthermore, there was no compromise in pre- and post-implanted catalase electrode sensitivity in ex vivo mouse brain. In vivo investigations performed in anaesthetised mice confirmed the ability of the H_2_O_2_ biosensor to detect increases in amperometric current following locally perfused/infused H_2_O_2_ and antioxidant inhibitors mercaptosuccinic acid and sodium azide. Subsequent recordings in freely moving mice identified negligible effects of control saline and sodium ascorbate interference injections on amperometric H_2_O_2_ current. Furthermore, the stability of the amperometric current was confirmed over a five-day period and analysis of 24-h signal recordings identified the absence of diurnal variations in amperometric current. Collectively, these findings confirm the biosensor current responds in vivo to increasing exogenous and endogenous H_2_O_2_ and tentatively supports measurement of H_2_O_2_ dynamics in freely moving NOD SCID mice.

## 1. Introduction

Reactive oxygen species (ROS), primarily superoxide (O_2_^−^) and hydrogen peroxide (H_2_O_2_), are produced routinely during cellular respiration and are homeostatically controlled by inherent antioxidant mechanisms. Increasing evidence has identified potentially toxic molecules, such as H_2_O_2_, as having a central role to play in normal physiological processes both at intracellular and extracellular levels. Over the last decade or so, widespread research has been carried out on the theory linking endogenous H_2_O_2_ to a neuromodulatory role in neurotransmitter release. In particular, extensive investigations undertaken by Rice and colleagues have identified intracellular regulation of neuronal activity and organelle function [1,2] coupled to extracellular neuron-glia signalling, synaptic transmission and plasticity functions [2,3,4] attributed to H_2_O_2_. However, despite this hypothesised neuromodulatory function, the molecule possesses the ability to be toxic when present at elevated concentrations and is associated with oxidative stress linking it to the pathophysiology of neurodegenerative diseases such as Parkinson’s disease (PD) [5,6,7]. Notwithstanding this, H_2_O_2_ itself is relatively unreactive and does not initiate oxidative damage unless exposed to free metal ions that can catalyse its conversion to the highly reactive hydroxyl radical [2]. Nevertheless, its basal concentrations are tightly controlled by a competing antioxidant network that functions to prevent tissue damage induced by ROS. These defence systems are thought to prevent ROS from causing irreparable damage by reacting with lipids, proteins and nucleic acids through enzymatic systems including glutathione peroxidase [8,9,10] and catalase [9,11].

Imbalanced ROS levels have been implicated and considered risk factors for a variety of human neurodegenerative disorders including PD. PD is a chronic age-related disorder characterised by progressive tremor, rigidity, bradykinesia, gait disturbance, postural instability and dementia [12]. Biochemical research has highlighted varying contributions to the aethiopathogenesis of PD from, amongst others, oxidative modifications, mitochondrial dysfunction and an impaired protein degradation system. One possible unifying molecular mechanism that can induce both the formation of protein inclusions and neuron degeneration is the oxidative reactions derived from the production of ROS [7]. The electron transport chain in the mitochondria is the principle source of ROS production following cellular respiration. Interruptions or disturbances in the electron transport chain allow electrons to be transferred and reduce molecular oxygen by one electron to form O_2_^−^, or two electrons to form H_2_O_2_ [7]. Moreover, the production of O_2_^−^ and H_2_O_2_ is not insignificant with as much as 5% of consumed O_2_ being converted into ROS [2]. Disturbances in normal mitochondrial function postulate a potentially pathological role for elevated H_2_O_2_ intracellularly. Moreover, elevated H_2_O_2_ can diffuse into the extracellular space exerting an adverse effect by perturbing its normal modulatory function on adjacent neurons.

For the aforementioned reasons, significant interest is emerging in the development of accurate and reliable tools that provide quantitative measurements of H_2_O_2_ dynamics in intact living brain tissue. In vivo amperometry is a particularly powerful tool that directly monitors extracellular fluid (ECF) compartments with excellent temporal and spatial resolution. Implanted amperometric sensors afford highly sensitive and selective real-time recordings that provide investigators with direct read-outs relating to particular neurochemical dynamics and fluctuations. Hitherto, limited electrochemical measurements of H_2_O_2_ in vivo have been reported, primarily due to the requirement of high overpotentials for the oxidation and reduction of the molecule severely hampering its selective detection [13]. Notwithstanding this, Kulagina et al. reported electrical stimulation evoked H_2_O_2_ increases in brain ECF using an implanted carbon microelectrode modified with a cross-linked redox polymer containing horse radish peroxidase [14]. More recently, Prussian blue enzyme stabilised in polydopamine was utilised by Li and colleagues enabling the selective detection of H_2_O_2_ in vivo during local microinfusions [15].

Herein, we utilise a recently described dual H_2_O_2_ biosensor originally designed by O’Brien and co-workers that incorporates individual platinum (Pt) cylinder electrodes for concurrent H_2_O_2_ detection and H_2_O_2_ degradation [16]. The total H_2_O_2_ current is obtained by subtracting the H_2_O_2_ degradation current from that recorded at the H_2_O_2_ detection electrode. The performance of this dual biosensor was further characterised by others both in vitro [17] and in the striatum of freely moving rats [18]. The principle objective of the work described within was to perform in vitro and ex vivo validation investigations on this dual H_2_O_2_ biosensor prior to an extensive in vivo characterisation study in the striatum of immunocompromised NOD SCID mice. We recently reported the characterisation of amperometric sensors for the real-time monitoring of nitric oxide and oxygen in the striatum of these mice [19]. The discovery of SCID mice was a milestone in the development of immunodeficient mice for xenotransplantation [20,21] and the generation of these so-called humanised mice can facilitate analysis of the underlying mechanisms of human disease pathogenesis. Furthermore, colleagues have generated and characterised a humanised mouse model of PD through xenotransplantation of induced pluripotent stem cell derived dopaminergic neurons into NOD SCID mouse striatum [22,23,24]. A prerequisite to the incorporation of the H_2_O_2_ biosensor into this humanised mouse model of PD is to validate the ability of this mice strain to facilitate reliable amperometric measurements over extended periods. This was achieved through a combination of acute and chronic recordings performed in anaesthetised and freely moving subjects respectively. To the best of our knowledge, this article is the first to detail the amperometric recording of H_2_O_2_ dynamics in the brain ECF of NOD SCID mice.

## 2. Materials and Methods

### 2.1. Chemicals and Solutions

All reagents utilised i.e., sodium chloride (NaCl; SigmaUltra), sodium hydroxide (NaOH; SigmaUltra), sodium hydrogen phosphate (NaH_2_PO_4_; A.C.S. reagent), potassium chloride (KCL; SigmaUltra), calcium chloride (CaCl_2_; SigmaUltra), magnesium chloride (MgCl_2_; SigmaUltra), hydrogen peroxide (H_2_O_2_; A.C.S. reagent 30.3%), ascorbic acid (AA; A.C.S. reagent), sodium azide (SA), mercaptosuccinic acid (MSA), sodium ascorbate, Nafion^®^ (5 wt % solution in a mixture of lower aliphatic alcohols and H_2_O), *o*-phenylenediamine (*o*-PD; 99+ %), catalase enzyme from bovine liver (CAT; lyophilised powder, 2000–5000 units/mg protein), and glutaraldehyde (Glu; grade 1, 25%) were purchased from Sigma Aldrich Chemical Co. (Dublin, Ireland). 

All in vitro calibrations were performed in phosphate buffered saline (PBS), pH 7.4; NaCl (0.15 M), NaOH (0.04 M) and NaH_2_PO_4_ (0.04 M) made up in doubly distilled deionised water. 

All in vivo compounds utilised in anaesthetised mice were dissolved in artificial cerebrospinal fluid (aCSF), pH 7.4; NaCl (0.15 M), KCl (0.004 M), CaCl_2_ (0.002 M) and MgCl_2_ (0.002 M) made up in doubly distilled deionised water. 

All in vivo compounds utilised in freely moving mice were dissolved up in 0.9% saline solution made up in doubly distilled deionised water. 

### 2.2. Amperometric H_2_O_2_ Biosensor

**Scheme 1:**
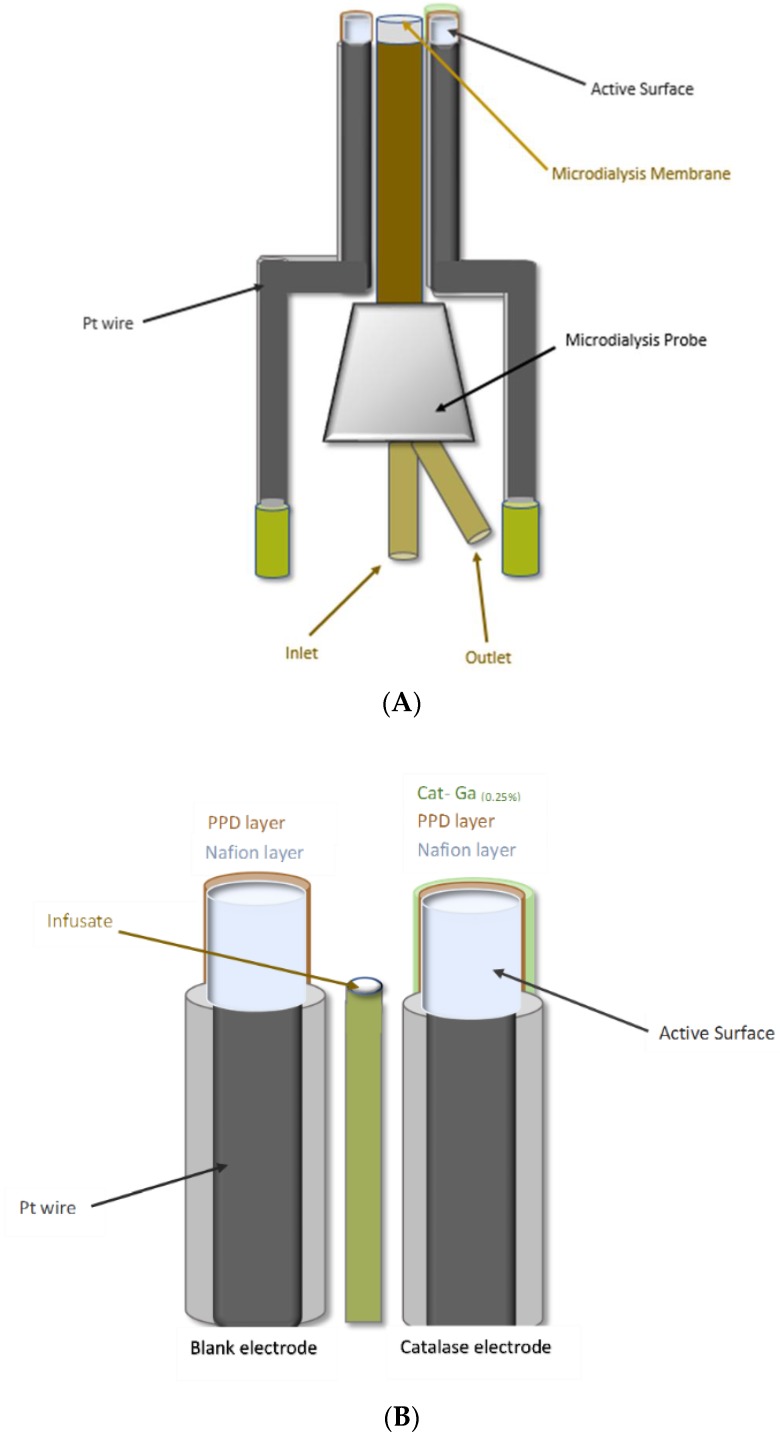
Graphical illustration of: (**A**) H_2_O_2_ biosensor/microdialysis probe construct (ca. 600–650 µm); and (**B**) H_2_O_2_ biosensor/microinfusion probe construct (ca. 250–300 µm).

Amperometric H_2_O_2_ recordings were performed using a dual sensor design, previously developed and characterised by other groups [16,17]. This design incorporates a Nafion^®^-poly-*o*-PD (PPD) coated 1 mm Pt cylinder electrode for H_2_O_2_ detection and a Nafion^®^-PPD-CAT-Glu coated 1 mm Pt cylinder electrode for H_2_O_2_ degradation. Both electrodes (see Scheme 1) are manufactured from Teflon^®^-insulated platinum/iridium (Pt/Ir 90%/10%) wire (127 µm bare diameter 5T, Science Products GmbH, Hofheim, Germany) and 1 mm cylinders were achieved by removing the Teflon^®^ from one end of the electrodes. For brevity, the dual biosensor design will be referred to as the H_2_O_2_ biosensor throughout the article. The total sensitivity of the H_2_O_2_ biosensor design was obtained by subtraction of the H_2_O_2_ degradation electrode (catalase) current from that recorded at the H_2_O_2_ detection electrode (blank) as previously reported by others [16,17,18]. O’Brien and colleagues describe this subtraction method as follows: once implanted in brain tissue, the currents measured at both the blank (*I_blank_*) and catalase (*I_catalase_*) electrodes will be comprised of both interference current (*I_INT_* and *I’_INT_*, respectively) from species present in the ECF and endogenous H_2_O_2_ (*I_HP_*) current:

*I_blank_ = I_INT_ + I_HP_*

*I_catalase_ = I’_INT_ + C_HP_ I_HP_*

∆*I = I_blank_ − I_catalase_*

where *C_HP_* is the proportion of undegraded H_2_O_2_ measured by the catalase electrode and ideally is zero. If the interference current determined during in vitro calibrations is similar at both blank and catalase electrodes, subtraction of the catalase current from the blank current will give rise to a current that depends only on H_2_O_2_. Furthermore, electrodes were initially paired based on their bare Pt H_2_O_2_ sensitivity prior to electrode surface modification [16].

Hitherto, the biosensor performance was confirmed in vitro by O’Brien et al. by determining the current responses to a myriad of electroactive interferents present in the brain ECF at concentrations representative of their physiological levels [16]. The biosensor demonstrated negligible responses from interfering species including AA, serotonin, DOPAC, dopamine, l-gluthathione, l-tyrosine, l-tryptophan, l-cysteine, 5-HIAA, homovanillic acid and uric acid. Subsequently, an extensive in vitro characterisation was undertaken by others that confirmed important physiological parameters including sufficient response time, limit of detection, negligible temperature and pH effects and sensocompatability confirmed over a 14-day period [17]. Furthermore, in vivo investigations in freely moving rats performed by the same group, support the efficacy of the sensor design by confirming the stability of the baseline current over a 12-day period. In addition, the surface integrity of the implanted H_2_O_2_ biosensor was confirmed by the systemic administration of the electroactive interferent, sodium ascorbate, causing negligible current deviations in striatal ECF [18]. Prior to implantation of the H_2_O_2_ biosensor in mouse striatum, matched selectivity was confirmed on both electrodes by calibrating against AA at a supraphysiological concentration which confirms membrane integrity.

### 2.3. In Vitro Calibrations

Calibrations were performed in a standard three-electrode glass electrochemical cell containing PBS electrolyte as has been previously described [25,26]. A saturated calomel electrode (SCE) acted as the reference electrode and a Pt rod utilised as the auxiliary electrode. Increasing concentrations of the respective analytes; i.e., H_2_O_2_ (standard calibration: 0–100 µM and extended calibration 0–10 mM), AA (0–1000 µM), SA (0–1000 µM) and MSA (0–1000 µM) were injected into the cell and the electrolyte solution was agitated using a magnetic stirrer for 2–3 s. The current was then measured under quiescent conditions after five minutes.

### 2.4. Ex Vivo Brain Tissue Investigations

Intact brains were removed from sacrificed NOD SCID mice and tissue homogenate was made up in PBS for sensocompatibility investigations. Separate sets of H_2_O_2_ biosensors were immersed in homogenate for 0, 1, 3, 7, and 14 days and H_2_O_2_/AA calibrations performed pre- and post-exposure. All H_2_O_2_ biosensors exposed to tissue homogenate were compared against their corresponding pre-exposed Day 0 calibrations. Furthermore, the physical effect of inserting the dip adsorbed catalase electrode component of the H_2_O_2_ biosensor was investigated in intact brains to mimic the effect of stereotaxic surgery implantation on the immobilised enzyme. This involved lowering the catalase electrode slowly into the intact brain using a micromanipulator (Narishige Inc., New York, NY, USA), leaving in place for 60 min and removing the electrode carefully. A H_2_O_2_ calibration was subsequently performed and comparisons were drawn against the sensitivity pre-insertion.

### 2.5. In Vivo Implantation and Surgery Protocol

NOD SCID mice (25–40 g) were housed in individually ventilated cages, with a maximum of five per cage in a temperature (17–23 °C), humidity and light controlled (12 h light, 12 h dark cycle) environment. Food and water were available ad libitum. H_2_O_2_ biosensors were implanted following a previously described procedure in NOD SCID mice [19] into the right or left striatum (*n* = 1/animal). For local investigations in anaesthetised mice, the H_2_O_2_ biosensor was co-implanted with either a microdialysis probe (2 mm membrane, BASi, Mt Vernon, IN, USA) or microinfusion probe (made in house). Coordinates used for all in vivo investigations, with the skull levelled between bregma and lambda, were as follows; A/P + 0.5, M/L ± 1.5 from bregma and D/V −4.5 from skull. Reference and auxiliary electrodes (8T Ag wires, 200 µm bare diameter) were placed in the cortex and soldered to a stainless steel support screw respectively. The electrodes were fixed to the skull with support screws and dental acrylate (Agnthos, Lidingö, Sweden). The mice were anesthetised with isoflurane anaesthesia (Abbott Laboratories, Dublin, Ireland), placed in a Stoelting stereotaxic frame (Stoeling Co., Wood Dale, IL, USA) and kept on a thermal pad with rectal probe (Stoeling Co., Wood Dale, IL, USA) to prevent hypothermia. A 10 mg·kg^−1^ subcutaneous (s.c) injection of the NSAID analgesic, Carprofen^®^ is administered at least 10 min prior to the incision in the scalp. Subsequently, Lidocaine analgesic (5 mg·kg^−1^) is applied topically to the incision site for local pain relief. Once surgery is complete, animals utilised for long term recordings are allowed to recover for a minimum of 24 h in a recording cage where they remain for the duration of the experiment. Alternatively, animals utilised for short term recordings under anaesthesia are sacrificed by cervical dislocation once recordings are ceased. All experimental procedures were performed under license AE19124/P010 in accordance with the European Communities Regulations 2002 (Irish Statutory Instrument 165/2013).

### 2.6. In Vivo Investigations and Experimental Conditions

Local and systemic administrations of characterisation compounds were undertaken in anaesthetised and freely moving animals respectively. Local delivery of compounds was facilitated through the combined implantation of the H_2_O_2_ biosensor with either a microdialysis or microinfusion probe (see Scheme 1). All local investigations in anaesthetised mice were carried out with the animal in the stereotaxic frame and constantly under isoflurane anaesthesia. Implanted amperometric electrodes were connected to a potentiostat through a six-pin Teflon socket and a bespoke screened four core cable manufactured in house. The desired potential (+700 mV vs. Ag wire) was applied to the H_2_O_2_ biosensor and the currents were allowed to stabilise prior to any local perturbation being performed. During experiments, the inlets of either microdialysis/microinfusion probes were connected to lightweight PEEK (BASi, Mt Vernon, IN, USA) tubing for the duration of administrations. The inlet tubing was connected to a 1 mL syringe (Hamilton, NV, USA) and perfused at specified flow rates using a Univentor-801 syringe pump (Agnthos, Lidingö, Sweden).

#### 2.6.1. Local Perfusions in Anaesthetised Mice Using Retromicrodialysis

This technique has been utilised previously to administer exogenous concentrations of the target analyte and characterisation compounds directly to the vicinity of the brain incorporating the implanted amperometric sensor in freely moving rats [18,27]. Briefly, each of the electrodes in the H_2_O_2_ biosensor design are lined up along the length of the microdialysis probe and temporally fixed in place using adhesive, taking extreme care not to disturb the modified electrode surfaces. The modified electrode surfaces are lined up adjacent to the sampling membrane on the microdialysis probe and once in close proximity, epoxy is gently spread along the length of the wire/probe interface to facilitate efficient fixation. The modified sensor tips are motioned carefully towards the sampling membrane using forceps over the course of the drying period. The combined sensor/probe construct is stored at 4 °C until surgical implantation.

Once implanted, local perfusions of H_2_O_2_ (0, 100 µM, 500 µM, 1 mM and 10 mM) and MSA (1 mM) occurred at a flow rate of 1.0 µL·min^−1^ using a syringe pump for 20 min, taking care to confirm perfusate exiting the probe outlet. A period of 15 min was left between each perfusion to allow currents to return to baseline prior to the subsequent administration. During the intermittent period, the solution was changed and pushed through the microdialysis tubing manually to wash out the previous concentration. The solution was then passed through the tubing at 1.0 µL·min^−1^ for a period to confirm flow, prior to reconnection to the probe inlet. All H_2_O_2_ perfusions were administered in a semi-randomised order. All MSA perfusions occurred subsequent to completion of H_2_O_2_ perfusions. A five-minute wash-out perfusion using aCSF was undertaken prior to MSA administration to mitigate against the effect of having residual H_2_O_2_ present within the probe following the previous perfusion.

#### 2.6.2. Local Infusions in Anaesthetised Mice Using Microinfusion

Investigations using combined H_2_O_2_ biosensor/microinfusion probes were utilised to compare and identify the most efficient method of local delivery. Microinfusion probes were manufactured by the following process developed in house. A 23G hypodermic needle (Aquilant Analytical Sciences, Dublin, Ireland) was cut to approximately 2 cm in length and the rough end smoothed with a metal file. An 8 cm length of deactivated fused silica tubing (110 µm i.d, 170 µm o.d, Aquilant Analytical Sciences, Dublin, Ireland) was passed through the needle, allowing ca. 1 cm and 5 cm to protrude from the smoothed and bevelled ends, respectively. The silica tubing was fixed in place using epoxy at both ends of the needle. Once dried, the silica tubing at the smoothed end was cut back flush to the needle. The tubing at the bevelled end was cut back to ca. 2 cm. and straightened. aCSF was then infused through the constructed probe using a microdialysis pump at a flow rate of 0.2 µL·min^−1^ and the infusate was confirmed at the end of the silica tubing. Subsequently, each of the electrodes in the H_2_O_2_ biosensor design are lined up along the length of the silica tubing and temporally fixed in place using adhesive, taking extreme care not to disturb the modified electrode surfaces. In this case, the modified electrode surfaces are lined up anterior to the end of the silica tubing as detailed in Scheme 1. When the modified electrode surfaces are in position, epoxy is gently spread along the length of the wire/silica tubing interface to facilitate efficient fixation. The modified sensor tips are motioned carefully in front of the silica tubing exit point using forceps over the course of the drying period. The construct is allowed to dry. Finally, the tip of a 1 mL plastic syringe (Aquilant Scientific, Dublin, Ireland) is cut and carefully placed up over the epoxied area until it reaches the bevelled needle tip. At this point, dental acrylate is dropped into this plastic component and allowed to solidify, acting as a method of fixation for the construct within the dental acrylate during surgical recordings. This latter step prevents the construct from sliding when attaching and reattaching the infusion tubing. 

Once implanted, local infusions of H_2_O_2_ (0, 20 µM, 50 µM, 100 µM, 1 mM and 10 mM), SA (1 mM) and MSA (1 mM) occurred at a flow rate of 0.2 µL·min^−1^ for 150 s. A period of 10 min was left between each infusion to allow currents to return and obtain a steady baseline prior to the subsequent administration. During the intermittent period, the solution was changed and pushed through the microdialysis tubing manually to wash out the previous concentration. The solution was then passed through the tubing at 0.2 µL·min^−1^ for a period to confirm flow prior to reconnection to the probe. All H_2_O_2_ infusions were administered in a semi-randomised order. All SA and MSA infusions occurred subsequent to completion of H_2_O_2_ infusions. A 150 s wash-out infusion using aCSF was undertaken prior to SA/MSA administration to mitigate against the effect of having residual H_2_O_2_ present within the probe following the previous infusion.

#### 2.6.3. Systemic Administrations in Freely Moving Mice

All long term amperometric recordings in freely moving mice were carried out with the animal in an open top recording cage. Implanted H_2_O_2_ biosensors were connected to a potentiostat through a six-pin Teflon^®^ socket and a bespoke screened four core cable manufactured in house which was mounted through a swivel slip ring (CPC Farnell, Preston, UK) above the subject’s head. This cable and swivel arrangement allowed free movement of the subject and permitted continuous amperometric recordings for up to five days at a time with an intermittent two-day disconnection period. The desired potential (+700 mV vs. Ag wire) was applied to the H_2_O_2_ biosensor and the currents were allowed to stabilise for a minimum of 24 h prior to any perturbation being performed. All solutions were prepared using 0.9% saline solution and administered in a volume of 1 mL·kg^−1^.

### 2.7. Instrumentation, Software and Data Analysis

In vitro and ex vivo constant potential amperometry (CPA) experiments were performed using a low-noise potentiostat (ACM Instruments, Cumbria, UK) and converted using an A/D converter (PowerLab, ADInstruments, Oxford, UK) running on a Dell computer. The CPA signal was recorded using LabChart software (v8, ADInstruments Oxford, UK). The in vivo amperometric current was detected using an eDAQ potentiostat (Quadstat, eDAQ Ltd, Sydney, Australia) and converted using an A/D converter (eCorder, eDAQ Ltd, Australia). The digital signal was then recorded using eChart software (v5.5, eDAQ Ltd, Sydney, Australia) running on a Dell Laptop. The highest gain setting was applied to the amperometric sensors during recordings to facilitate the most efficient signal to noise ratio. Signal processing was performed on collected data to further improve signal to noise ratios using the eChart software. All data analysis was performed using GraphPad Prism v5 (GraphPad Software Inc., San Diego, CA, USA). 

As alluded to in Section 2.2, if the interference current determined during in vitro H_2_O_2_ calibrations is similar at both blank and catalase electrodes, subtraction of the catalase current from the blank current will give rise to a current that depends selectively on H_2_O_2_. To guarantee accuracy with this subtraction method, all in vivo figures presented had their baseline currents normalised to 100% illustrating the overall subtraction in current as a percentage of the pre-perturbation baseline current. The respective baselines currents were calculated by averaging the current over a defined period prior to the perturbation i.e., 2.5 min for local administrations and 10 min for systemic administrations. This removes any discrepancies that may exist between in vivo baseline levels between animals by ensuring all current changes are normalised and relative to that particular sensor. The significance of differences observed was estimated using the Student’s *t*-test for paired or unpaired observations where appropriate. One-way ANOVAs followed by Bonferroni post hoc test were performed where appropriate. Two-tailed levels of significance were used with *p* < 0.05 considered to be significant. All data is presented as mean ± standard error (SEM), with *n* = number of animals except for 24 h recordings where *n* = number of days or nights/number of animals. One H_2_O_2_ biosensor was implanted per animal.

## 3. Results

### 3.1. In Vitro Investigations on H_2_O_2_ Biosensor Design

**Figure 1:**
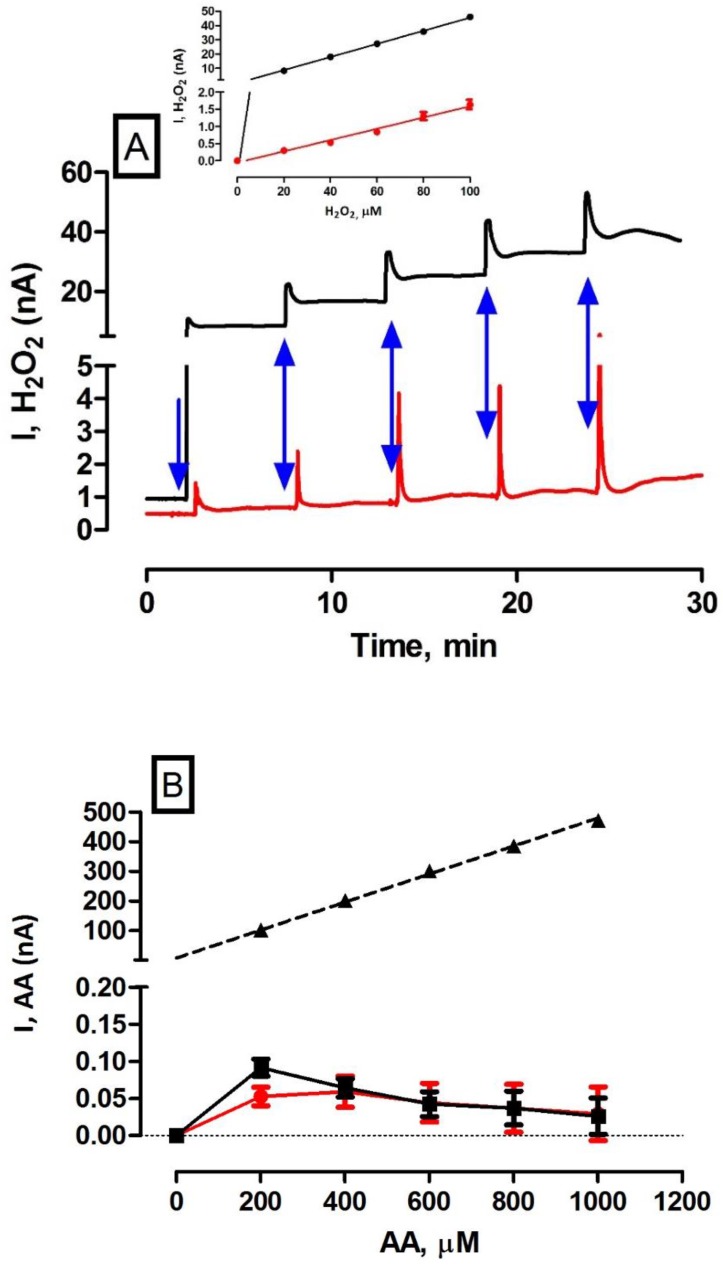
(**A**) Typical in vitro data obtained for a 0 to 100 µM H_2_O_2_ calibration using H_2_O_2_ blank electrode (black trace) and catalase electrode (red trace). Blue arrows indicate addition of 20 µM H_2_O_2_ aliquots. *Inset:* Current concentration profiles for H_2_O_2_ calibration on H_2_O_2_ blank electrode (*n* = 39, *r*^2^ = 0.99, black trace) and H_2_O_2_ catalase electrode (*n* = 39, *r*^2^ = 0.99, red trace). (**B**) Current concentration profiles for AA calibration (0–1000 µM) on H_2_O_2_ blank electrode (*n* =12, black trace), H_2_O_2_ catalase electrode (*n* = 12, red trace) and unmodified bare platinum (Pt) electrode as comparison (*n* = 24, *r*^2^ = 0.99, dashed trace). All concentration profile data are presented as mean ± SEM.

Initially, the principle objective was to demonstrate the functionality and reproducibility of the previously characterised [16,17] amperometric H_2_O_2_ biosensor in vitro. Calibrations carried out using H_2_O_2_ blank electrodes demonstrated excellent linearity over 0–100 µM, *r*^2^ = 0.99 (see Figure 1A) and a recorded sensitivity of 459 ± 6 pA·µM^−1^ (*n* = 39). This sensitivity value is similar to that recorded previously for the same sensor type by other researchers [17]. Calibrations undertaken on the catalase electrode yielded excellent linearity (*r*^2^ = 0.99, see Figure 1A) over the same calibration range and a sensitivity value of 16 ± 1 pA·µM^−1^ (*n* = 39). Again, this sensitivity corroborated previous work performed on this sensor type [17]. The net subtraction of the catalase electrode current from the blank electrode current in vivo yields the total current attributed to H_2_O_2_ by removing any contribution that may be attributed to electroactive interferents. Therefore, the sensitivity of the subtracted paired design was 443 ± 6 pA·µM^−1^ (*n* = 39). Furthermore, there was no significant difference between the blank and paired sensitivities (*p* = 0.06) and the catalase sensor has a negligible contribution to the overall H_2_O_2_ sensor response (ca. 97% degradation). 

As detailed previously, if the interference current determined during in vitro calibrations is similar at both blank and catalase electrodes, subtraction of the catalase current from the blank current will give rise to a current that depends only on H_2_O_2_. The selectivity of both catalase and blank sensors has been reported previously with negligible contribution from a myriad of interfering species [16]. Due to its high abundance in brain ECF, H_2_O_2_ biosensors are tested against AA to confirm selectivity prior to in vivo implantation. The AA rejection characteristics of both blank and catalase sensors are comparable to previous work reported [17]. The current-concentration profile in Figure 1B illustrates the self-blocking capability of the PPD/Nafion^®^ rejection layer. The effect of physiological (400 µM) and supraphysiological (1000 µM) AA concentrations were examined. The current response of the blank H_2_O_2_ sensor to 400 µM and 1000 µM AA is 64 ± 12 pA (*n* = 12) and 26 ± 24 pA (*n* = 12), respectively. At lower concentrations, i.e. 200–400 µM, a limited amount of AA is transported across the polymer membrane. This feature diminishes with subsequent injections as the polymer pore size self-blocks the diffusion of incoming AA due to the entrapment of AA and its oxidised metabolite dehydroascorbic acid (DHA) within the PPD polymer matrix. This has been reported previously and is characteristic of PPD polymers [25,28]. Similarly, the corresponding currents of the catalase H_2_O_2_ sensor to 400 µM and 1000 µM AA are 59 ± 21 pA (*n* = 12) and 29 ± 36 pA (*n* = 12) respectively. No significant difference was observed between H_2_O_2_ blank and H_2_O_2_ catalase electrodes for their corresponding currents at 400 µM (*p* = 0.84) and 1000 µM (*p* = 0.94). The subtracted currents of the paired sensor are 5 ± 24 pA (400 µM) and −3 ± 44 pA (1000 µM). These findings confirm the in vitro selectivity of the paired sensor for H_2_O_2_. For comparative purposes, Figure 1B includes the current concentration profile for an AA calibration (0–1000 µM) on an unmodified Pt electrode (*n* = 24). The response is linear (*r*^2^ = 0.99) and the corresponding currents at 400 µM and 1000 µM are 203 ± 10 nA and 471 ± 13 nA respectively. This clearly illustrates the efficacy of the PPD/Nafion^®^ layer at blocking this ubiquitous interferent and confirming the performance of the permselective membrane. 

### 3.2. Ex Vivo Sensocompatibility Investigations on H_2_O_2_ Biosensor Design

**Figure 2:**
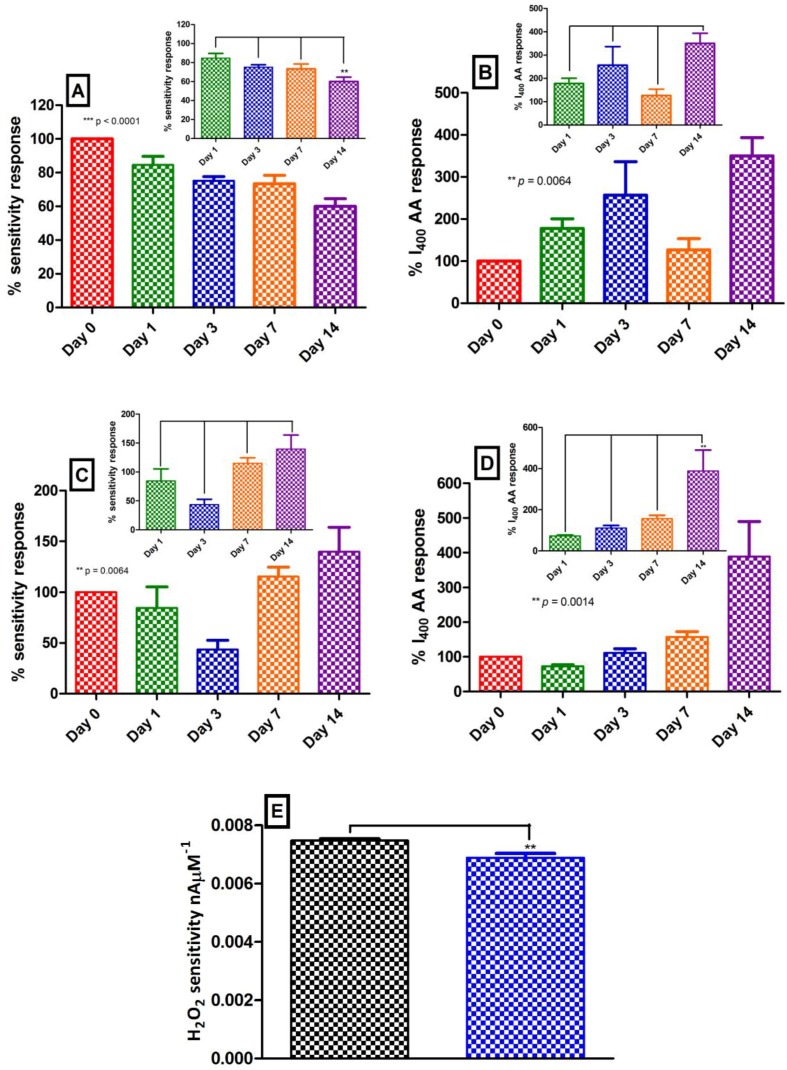
Ex vivo studies detailing effect of brain tissue (BT) exposure (0 – 14 day, *n* = 4) on: (**A**) H_2_O_2_ blank electrode sensitivity; (**B**) H_2_O_2_ blank electrode selectivity; (**C**) H_2_O_2_ catalase electrode sensitivity; and (**D**) H_2_O_2_ catalase electrode selectivity. *Insets:* Days 1–14 post hoc analysis. Data represented as mean ± SEM. (**E**) Effect of insertion/removal of catalase electrode into mouse brain ex vivo (*n* = 12). Catalase electrode sensitivity pre-insertion (black) vs. catalase electrode sensitivity following removal (blue). Sensitivity data represented as mean ± SEM.

Sensocompatibility investigations were performed ex vivo with the objective of trying to mimic the conditions encountered by the H_2_O_2_ biosensor in vivo. This is a vital component of the biosensors characterisation since implantation in mouse brain exposes the biosensor to a harsh biological milieu that contains electrode poisons such as lipids and proteins which impact on performance. Furthermore, the tissue matrix surrounding the biosensor surface both restricts mass transport of the target analyte to the electrode surface and reacts physiologically to the presence of the sensor. This is a well-documented phenomenon for in vivo electrochemical measurements [26,29,30] that warrants considerable attention. Although not an exact representation of the physiological environment, exposing an electrochemical sensor to brain tissue homogenate serves as a good indication of the effect of in vivo conditions on the biosensors performance. Figure 2A,C highlight the effect of brain tissue exposure on the respective sensitivities of H_2_O_2_ blank and H_2_O_2_ catalase electrodes with the sensitivity of each electrode expressed as a percentage of its pre-exposure sensitivity (Day 0). Furthermore, Day 0 sensitivities are expressed as 100%. In detail, H_2_O_2_ blank electrodes (see Figure 2A) demonstrated a significant difference (*p* < 0.0001, one-way ANOVA, *n* = 4) across Day 0 (100%), Day 1 (85 ± 5%), Day 3 (75 ± 2%), Day 7 (73 ± 5%) and Day 14 (60 ± 4%). This initial drop in sensitivity has been reported by numerous groups [17,31] upon contact with physiological tissue and is well within the adequate expected range [27]. Bonferroni post hoc analysis identified that Day 14 differed significantly from Day 1 (*p* < 0.01), however, no significant difference was observed among Day 1, Day 3 and Day 7 (see Figure 2A inset). Similarly, H_2_O_2_ catalase electrodes (see Figure 2C) demonstrated a significant difference (*p* < 0.01, one-way ANOVA, *n* = 4) across Day 0 (100%), Day 1 (85 ± 21%), Day 3 (44 ± 9%), Day 7 (115 ± 9%) and Day 14 (140 ± 24%). Intuitively, one would interpret the H_2_O_2_ catalase electrode response in a similar manner to the H_2_O_2_ blank electrode, however, it is important to remember that the former functions as a quantitative measurement of H_2_O_2_ degradation. Therefore, Figure 2C identifies an initial improvement in performance on Day 1 and Day 3 through a decrease in H_2_O_2_ detected at the electrodes surface. However, interpretation of these findings in parallel with H_2_O_2_ blank electrodes postulates a reduction in analyte detection occurring as a result of inhibited diffusion across the biosensor membrane through adsorption of proteins and lipids within the tissue homogenate. It is imperative not to confuse this with an improvement in biosensor performance. Bonferroni post hoc analysis identified no significant difference (*p* > 0.05) between Day 1 exposure and the subsequent Days 3–14 (see Figure 2C inset). 

It is also important to perform sensocompatibility investigations on the AA rejection characteristics of the permselective membrane. In this case, the currents obtained for brain tissue exposed electrodes at 400 µM and 1000 µM AA are expressed as a percentage of their corresponding pre-exposed current (Day 0). Furthermore, Day 0 currents are expressed as 100%. In summary, H_2_O_2_ blank electrodes (see Figure 2B) demonstrated a significant difference (*p* < 0.01, one-way ANOVA, *n* = 4) across Day 0 (100%), Day 1 (178 ± 23%), Day 3 (257 ± 80%), Day 7 (127 ± 27%) and Day 14 (350 ± 43%) for 400 µM AA. Further, a significant difference (*p* < 0.05, one-way ANOVA, *n* = 4) across Day 0 (100%), Day 1 (224 ± 32%), Day 3 (384 ± 143%), Day 7 (150 ± 46%) and Day 14 (469 ± 79%) at 1000 µM AA was also noted. Bonferroni post hoc analysis identified no significant difference (*p* > 0.05) between Day 1 exposure and the subsequent Day 3–14 at both 400 µM AA (see Figure 2B inset) and 1000 µM AA (data not shown). Again, care must be taken when interpreting these results since an increase in detection across the days signifies a compromise in membrane integrity, nevertheless, this would be expected following exposure to proteins and lipids as has been previously reported [26]. Despite this slight increase in AA detection, the currents recorded at both concentrations of AA are <1% of the bare Pt response detailed in the previous section. The extracellular levels of AA are reported to be between 200–500 µM [32] and Figure 1B illustrates that the self-blocking characteristic of the PPD polymer occurs within this concentration range. A limited amount of AA is transported across the polymer membrane which dissipates with further injections due to the polymer sterically hindering incoming AA due to the entrapment of AA and DHA within the PPD polymer matrix. We hypothesised previously that this phenomenon occurs in vivo causing the PPD layer to self-block after initial exposure to AA and remain so for the duration of the implantation [26] mitigating against its potential as an interfering compound.

Similarly, H_2_O_2_ catalase electrodes (see Figure 2D) demonstrated a significant difference (*p* < 0.01, one-way ANOVA, *n* = 4) across Day 0 (100%), Day 1 (73 ± 5%), Day 3 (111 ± 12%), Day 7 (157 ± 16%) and Day 14 (389 ± 101%) for 400 µM AA. Further, a significant difference (*p* < 0.05, one-way ANOVA, *n* = 4) across Day 0 (100%), Day 1 (83 ± 7%), Day 3 (129 ± 19%), Day 7 (192 ± 23%) and Day 14 (556 ± 195%) at 1000 µM AA was also noted. Bonferroni post hoc analysis identified that Day 14 differed significantly from Day 1 (*p* < 0.01), however, no significant difference was observed among Day 1, Day 3 and Day 7 for both 400 µM (see Figure 2D inset) and 1000 µM (data not shown). Collectively, these findings from the H_2_O_2_ biosensor indicate that the biosensors performance remains stable during Days 1–7 of tissue homogenate exposure, beyond this some uncertainty exists concerning H_2_O_2_ blank electrode sensitivity and H_2_O_2_ catalase electrode selectivity. These assumptions will be revisited later during chronic in vivo recordings. 

Another important consideration that warrants investigating is the physical effect that in vivo implantation has on the adsorbed enzyme on the H_2_O_2_ catalase electrode. This was best examined ex vivo due to difficulties encountered when trying to remove implanted sensors from sacrificed mice. In vivo implantation conditions were mimicked by inserting pre-calibrated H_2_O_2_ catalase electrodes (*n* = 12) into mouse brains using a micromanipulator and removing the electrodes following 60 min exposure. A second calibration was performed and the sensitivities compared against pre-implantation. It is obvious from Figure 2E that no compromise in sensitivity was identified as a result of the physical effect of lowering the electrodes into mouse brain tissue. Moreover, a significantly reduced (*p* < 0.01) sensitivity was recorded for the post-implanted (6.9 ± 0.5 pA·µM^−1^, *n* = 12) H_2_O_2_ catalase electrodes compared to the pre-implanted (7.5 ± 0.2 pA·µM^−1^, *n* = 12). This finding corroborates Figure 2C were an initial reduction in sensitivity is observed over the first 72 h. We postulated that a reduction in analyte detection occurring as a result of inhibited diffusion across the biosensor membrane through adsorption of proteins and lipids within the tissue homogenate and not as an improvement in enzyme activity. Nevertheless, these findings confirm retention of adsorbed enzyme on implanted H_2_O_2_ catalase electrodes supporting their functionality during in vivo recordings.

### 3.3. Effect of Acute Administrations on H_2_O_2_ Current in the Striatum of Anaesthetised NOD SCID Mice 

#### 3.3.1. Combined Amperometry and Retromicrodialysis Investigations

**Figure 3:**
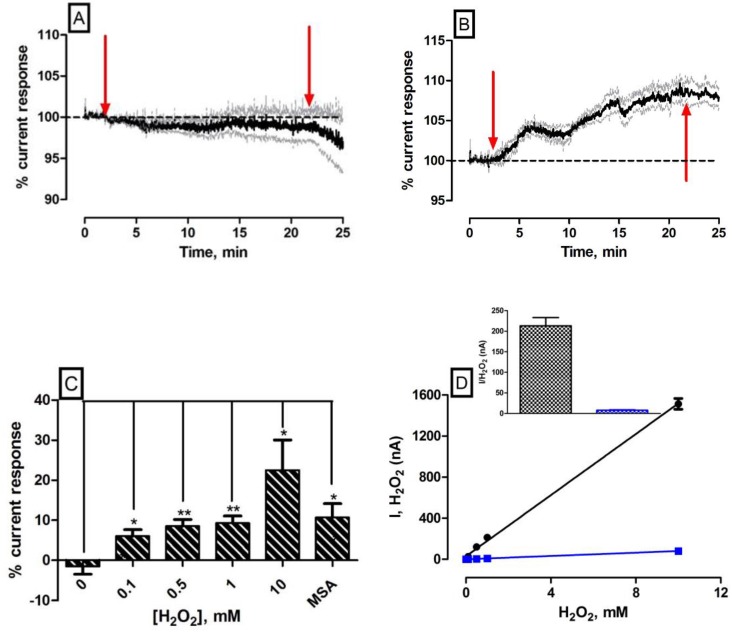
Averaged raw data percentage response of subtracted current from H_2_O_2_ biosensor co-implanted with a microdialysis probe in the striatum of anaesthetised NOD SCID mice (*n* = 4) to: (**A**) perfusion of artificial cerebrospinal fluid (aCSF); and (**B**) retroperfusion of 1 mM H_2_O_2_/aCSF. Red arrows indicate start/end of 20-min perfusion. Mean percentage current response represented by black trace, percentage error represented by grey trace. (**C**) Dose effect of retrodialysis of 0–10 mM H_2_O_2_/aCSF (*n* = 4) and 1 mM mercaptosuccinic acid (MSA)/aCSF (*n* = 3) on subtracted H_2_O_2_ current. Percentage current response data represented as mean ± SEM. (**D**) In vitro current concentration profiles for extended H_2_O_2_ calibration (0–10 mM) on H_2_O_2_ blank electrode (*n* = 4, *r*^2^ = 0.99, black trace) and catalase electrode (*n* = 3, *r*^2^ = 0.99, blue trace). *Inset:* Comparison of average currents for both electrodes at 1 mM H_2_O_2_. All concentration profile data is presented as mean ± SEM. * denotes level of significance.

As we alluded to earlier, local perfusion through co-implanted microdialysis probes has been utilised previously to administer exogenous concentrations of the target analyte and characterisation compounds directly to the vicinity of the brain incorporating the implanted amperometric sensor in freely moving rats [18,27]. The experiments described within this body of work using this characterisation technique were performed exclusively in anaesthetised mice. The principal justification being that the mouse model is not suitable to combine microdialysis probes and amperometric sensors in awake subjects due to obvious size constraints. All local administrations occurred using aCSF as the vehicle; therefore, it was imperative to investigate the effect of the control perfusate on the subtracted amperometric signal. Figure 3A illustrates the effect of a 20-min perfusion on the amperometric signal (see Supplementary Material Figure S1A,B for individual electrode responses following perfusion). The subtracted amperometric current ∆I (−73 ± 80 pA, *n* = 4) was not significantly different (*p* = 0.49) from pre-perfusion levels and corresponded to a percentage change of −2 ± 2%. In Supplementary Figure S1B, it is obvious that both electrode currents decreased from baseline over the course of the perfusion. This was attributed to the net diffusion of endogenous species located around the microdialysis probe across the membrane and into the perfusate/dialysate. Notwithstanding this, the overall subtracted current only deviated slightly from pre-perfusion levels indicating a negligible effect of control administrations on the amperometric response. The current returned towards the pre-perfusion baseline level upon cessation of the perfusate (data not shown).

In contrast, the retroperfusion of 1 mM H_2_O_2_/aCSF resulted in a significant increase (*p* < 0.05) in current (90 ± 34 pA, *n* = 4) which translated into a percentage change of 9 ± 2% compared to pre-perfusion baselines (see Figure 3B). Figure 3B highlights the effect of a 20-min retroperfusion of 1 mM H_2_O_2_/aCSF on the amperometric signal (see Figure S2A,B for individual electrode responses) that was significantly different when compared against aCSF control. There is an obvious increase in subtracted amperometric current albeit at a much lower level than was expected. Figure 3C and Table 1 detail the in vivo characterisation data obtained across all local perfusions in the striatum of anaesthetised mice with significant difference observed against control aCSF perfusion for each of the concentrations administered. The local delivery of varying concentrations of H_2_O_2_ to the site of the H_2_O_2_ biosensor confirms its ability to detect exogenous analyte. However, it is apparent from Figures S1 and S2 that an increase in the H_2_O_2_ detecting electrode (H_2_O_2_ blank) of the dual biosensor only occurs at a concentration ≥1 mM which is contradictory to what we would expect. In vitro calibrations performed over an extended calibration range (see Figure 3D) confirm that the H_2_O_2_ blank electrode demonstrates a current change of 213 ± 20 nA (*n* = 4) at this concentration range (see Figure 3D inset). These findings identify a lack of correlation between currents measured in vitro and those measured in vivo using this retromicrodialysis technique.

It is critical to validate the ability of the biosensor to measure endogenous levels of H_2_O_2_ present within the striatum. The local delivery of the glutathione peroxidase inhibitor MSA was investigated to determine the H_2_O_2_ biosensors ability to measure alterations in the concentration of endogenously available H_2_O_2_. Glutathione peroxidase is considered to be an extremely important antioxidant enzyme in the brain with high neuroprotective characteristics [10,33] primarily attributed to the degradation of H_2_O_2_ [8,9,10]. Local perfusions of MSA resulted in a significant increase (*p* < 0.05) in subtracted amperometric percentage current response (11 ± 3%, *n* = 3), compared against control aCSF response recorded at the biosensor surface supporting this assumption (see Figure 3C, Figure S2E and F and Table 1). This supports work undertaken by others whereby the local perfusion of MSA into the striatum of freely moving rats produced an increase in current measured by the amperometric H_2_O_2_ biosensor [18]. Furthermore, Li and colleagues reported that local infusion of MSA in anaesthetised rats resulted in an increase in electrochemical H_2_O_2_ current measured at their polymer modified microelectrode surface [15]. 

As alluded to previously, there is considerable discrepancy and lack of correlation observed between the maximum current responses in vivo using the retromicrodialysis technique and those measured in vitro. Although not entirely unexpected, the larger concentrations of H_2_O_2_ (1 mM and 10 mM) were anticipated to yield larger responses than those detected (see Table 1). Other groups have reported similar findings using the H_2_O_2_ biosensor in the extracellular fluid of rats [18]. There are a number of limitations associated with the combined microdialysis/biosensor construct that may impact on our findings. For example, a large trauma layer can be produced around the implanted microdialysis probe that restricts transport of the perfused analyte to the electrode surface [34,35,36]. The presence of tissue damage following implantation of the probe/biosensor construct raises serious questions about how that damage may either influence the measurement process or the localised physiology that governs analyte flux to the sensor surface [37]. Furthermore, variations in the relative recovery of microdialysis probes can lead to significant under estimations in the concentration of recovered/retroperfused analytes [18,37,38,39]. Tissue tortuosity and the highly efficient antioxidant mechanisms present in the mouse brain would also have significant impact on the amount of H_2_O_2_ diffusing to the biosensor surface. 

#### 3.3.2. Combined Amperometry and Microinfusion Investigations

**Figure 4:**
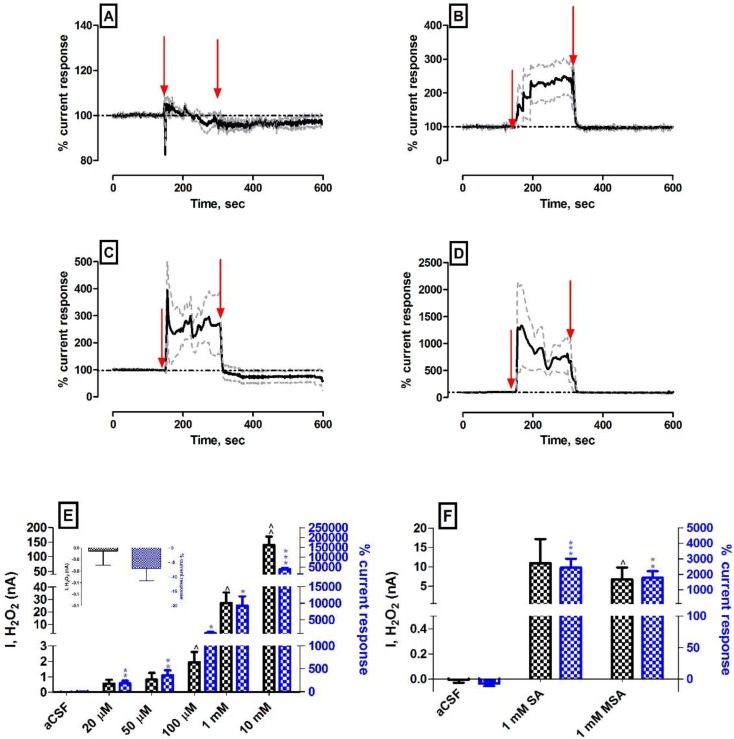
Averaged raw data percentage response of subtracted current from H_2_O_2_ biosensor co-implanted with a microinfusion probe in the striatum of anaesthetised NOD SCID mice (*n* = 5) to: (**A**) infusion of aCSF; (**B**) infusion of 20 µM H_2_O_2_/aCSF; (**C**) infusion of 50 µM H_2_O_2_/aCSF; and (**D**) infusion of 100 µM H_2_O_2_/aCSF. Red arrows indicate start/end of 150-s infusion. Mean percentage current response represented by black trace, percentage error represented by grey trace. Dose effect of infusion of: (**E**) 0–10 mM H_2_O_2_/aCSF (*n* = 5); and (**F**) 1 mM sodium azide (SA) and MSA (*n* = 3) on subtracted H_2_O_2_ current. Current (left *y*-axis) and percentage current response (right *y*-axis) data represented as mean ± SEM. ^ and * denote level of significance for currents and percentage currents respectively.

A separate set of acute investigations were devised in vivo to try elucidate this concept further. The microdialysis probes were replaced with microinfusion probes to address the aforementioned concerns. Moreover, the infusate will not be affected by relative recovery concerns and the size of the construct is reduced considerably (see Scheme 1 in materials and methods). It is important to highlight that the infusion time is reduced to ca. 150 s at a flow rate of 0.2 µL·min^−1^ to avoid issues with larger volumes of fluid entering the mouse striatum. Figure 4A illustrates the effect of a 150-s infusion of aCSF on the amperometric signal (see Figure S3A for individual electrode responses following infusion). The subtracted amperometric current ∆I (−5 ± 24 pA, *n* = 5) was not significantly different (*p* = 0.18) from pre-perfusion levels and corresponded to a percentage change of −7 ± 4%. This decrease in amperometric current can be attributed to the drift in baseline current observed during the infusions which is associated with the relatively short settling period afforded to acute experiments. Figure S3A clearly details no current change following control infusions. Figure 4B–D and Table 2 detail the effect of local infusions of 20 µM, 50 µM and 100 µM H_2_O_2_ on subtracted biosensor current. The maximum current measured for 20 µM: 0.56 ± 0.25 nA (*n* = 5), 50 µM: 0.82 ± 0.44 nA (*n* = 5) and 100 µM: 1.94 ± 0.68 nA (*n* = 5) demonstrated significant differences (*p* < 0.01, *p* < 0.01 and *p* < 0.05) against aCSF controls and corresponded to percentage changes of 184 ± 56%, 359 ± 107% and 1941 ± 675% respectively from pre-infusion baselines. Figures S3–S5 highlight the individual electrode responses following all infusions and it is apparent that all doses of H_2_O_2_ produce an increase in amperometric currents which is in stark contrast to the retromicrodialysis investigations. 

It is obvious from these findings and those detailed in Table 2 that the incorporation of a microinfusion probe into the construct design has mitigated against the limitations identified with the previous acute investigations. A robust and reproducible response from the H_2_O_2_ biosensor to low levels of infused analyte (20–100 µM) is in stark contrast to the retromicrodialysis investigations. Moreover, the H_2_O_2_ blank electrode demonstrated instantaneous responses to these levels which was absent previously. A plausible explanation for the improved results is that local infusion of H_2_O_2_ guarantees that the total concentration in the infusate reaches the site of implantation, alleviating against fluctuating probe recovery rates associated with retromicrodialysis. Furthermore, the smaller dimensions of the probe ensure the H_2_O_2_ biosensor active surfaces are in closer proximity to the silica outlet, reducing: (1) the time required for the analyte to reach the sensor surface; and (2) the time allowed for the endogenous antioxidant mechanism to exert its function. The larger concentrations of H_2_O_2_ (1 mM and 10 mM) followed this trend and resulted in large increases as detailed in Table 2 and Figures S4,5. Linear regression analysis performed on the infusion data in Table 2 identified a tentative in vivo slope/sensitivity value of 13.8 ± 0.7 pA·µM^−1^ (*n* = 5). This differs significantly (*p* < 0.001) from the in vitro sensitivity value of 443 ± 6 pA·µM^−1^ (*n* = 39) but care must be taken when interpreting this data. In vitro investigations ensure experimental reproducibility due to uniform conditions which cannot be guaranteed in vivo. Moreover, tissue heterogeneity may cause the H_2_O_2_ to diffuse through the extracellular space with a tortuous path [37], coupled with the aforementioned plethora of inherent antioxidant mechanisms, can significantly impact on the actual concentration that is detected at the electrode surface. Nevertheless, it provides a valuable indication of the biosensors ability to measure low levels of H_2_O_2_ in the striatum of the mouse. 

In line with the previous acute investigations, it was imperative to investigate the ability of the biosensor to measure endogenous levels of H_2_O_2_ present within the striatum through inhibition of the highly efficient antioxidant mechanism. The local delivery of the glutathione peroxidase inhibitor MSA demonstrated significant differences against control aCSF perfusions and was utilised once more. In addition, the catalase inhibitor SA was investigated to determine the effect of an alternative enzymatic mechanism on the H_2_O_2_ biosensor current response. Catalase is a well acknowledged antioxidant which detoxifies H_2_O_2_ by reducing it to molecular oxygen and water [11,40]. Significant increases (*p* < 0.001 and *p* < 0.01) in subtracted amperometric current (10.96 ± 6.19 nA, *n* = 3 and 6.75 ± 3.10 nA, *n* = 3) and percentage current responses (2439 ± 558%, *n* = 3 and 1782 ± 422%, *n* = 3) for SA and MSA respectively, compared against control aCSF response, were recorded at the biosensor surface. These findings support measurement of increasing H_2_O_2_ concentrations following enzymatic inhibition (see Figure 4F and Figure S4C,D and Table 2). It was critical that we addressed any concerns around false positives generated as a result of the electrochemical detection of SA and MSA at the H_2_O_2_ sensor surfaces. Therefore, 0–1000 µM in vitro SA and MSA calibrations were performed on H_2_O_2_ blank electrodes. Figure S6A,B illustrates negligible interference from both compounds on the H_2_O_2_ blank electrode at this concentration which supports the assumption that the increase in current observed during the infusions is due to increased endogenous H_2_O_2_ detection. As described previously, a 150-s wash-out infusion using aCSF was undertaken prior to SA/MSA administration to mitigate against the effect of having residual H_2_O_2_ present within the probe following the previous infusion. The effect of exposing the H_2_O_2_ blank electrode to such high concentrations of analyte was also investigated to confirm retention of SA and MSA rejection characteristics. Figure S6C illustrates no effect on the rejection characteristics of the H_2_O_2_ blank electrodes following exposure to such high concentrations of H_2_O_2_ (0–10 mM). Taken collectively, these findings strongly support the increase in H_2_O_2_ biosensor current occurring as a result of increasing endogenous concentrations and not as a result of the electrochemical detection of the infused compound. Similar increases in H_2_O_2_ concentrations have been reported following SA [9,41] and MSA [15,18] administrations by others that further corroborate our findings. 

### 3.4. Chronic Investigations in Freely Moving NOD SCID Mice

#### 3.4.1. Effect of Control Saline Injections on H_2_O_2_ Current in the Striatum of Freely Moving NOD SCID Mice

**Figure 5:**
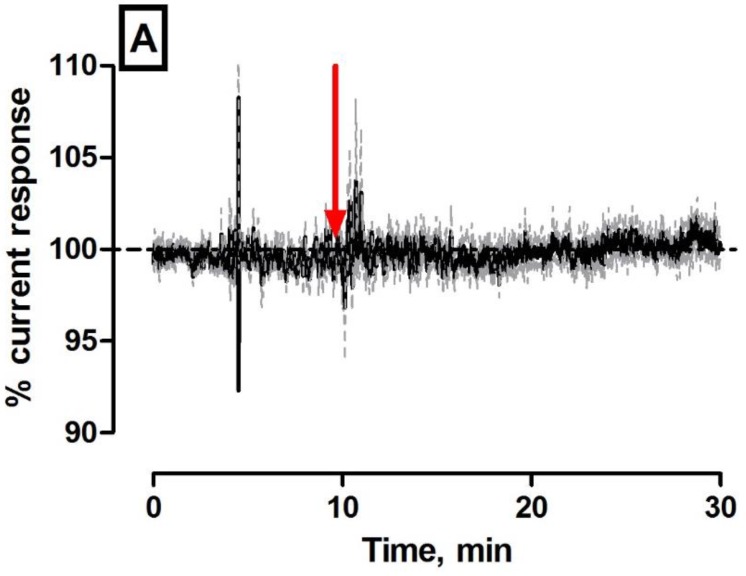

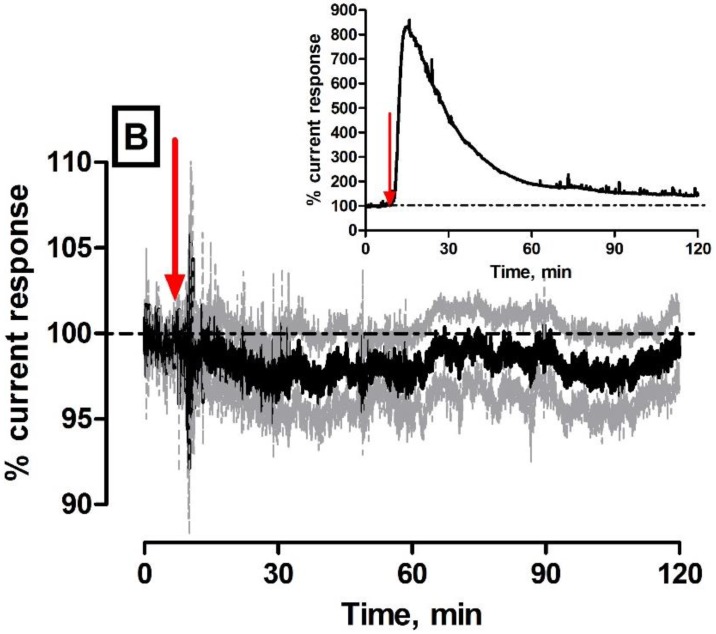
Averaged raw data percentage response of subtracted amperometric current from H_2_O_2_ biosensor implanted in the striatum of NOD SCID mice to: (**A**) 1 mL·kg^−1^ injection of 0.9% saline (*n* = 6); and (**B**) 500 mg·kg^−1^ injection of sodium ascorbate (*n* = 5). *Inset:* Typical example of raw data percentage response of a bare Pt 1 mm cylinder electrode implanted in the striatum of a NOD SCID mouse to 500 mg·kg^−1^ injection of sodium ascorbate. Red arrow indicates point of injection. Mean percentage current response represented by black trace, percentage error represented by grey trace.

In vivo characterisation compounds utilised during long term recordings were prepared using 0.9% saline solution and administered by intraperitoneal injection in a volume of 1 mL·kg^−1^. Therefore, it was imperative to characterise the sensor response to control saline injections. The averaged raw data current response following saline administration is detailed in Figure 5A (*n* = 6). Briefly, the subtracted current from the H_2_O_2_ biosensor was 35 ± 17 pA which corresponds to a 1 ± 2% deviation in amperometric current that reached a maximum response after 4 ± 1 min and returned to basal levels after 9 ± 2 min. Similar short-lived transient effects following control saline injections have been reported previously using amperometric sensors in the striatum of freely moving mice [19] and rats [27,42]. 

#### 3.4.2. Effect of Sodium Ascorbate Interference Injections on H_2_O_2_ Current in the Striatum of Freely Moving NOD SCID Mice

Previously reported work has demonstrated that H_2_O_2_ blank and catalase electrodes possess excellent selectivity towards H_2_O_2_ in vitro against a wide range of electroactive interferents (e.g., ascorbic acid, dopamine, DOPAC, uric acid, and serotonin) found endogenously in brain extracellular fluid [16]. It is a vital step in the characterisation process to confirm that the selective membrane has not degraded when placed in the in vivo environment and that the H_2_O_2_ biosensor exhibits similar selectivity characteristics to those recorded in vitro. Furthermore, the functionality of the paired sensor depends on matched interference between both sensors as detailed earlier. With its high concentration and ease of oxidation, ascorbate is the most readily detectable molecule in brain ECF using in vivo amperometry [32,43]. For these reasons it was important to investigate the effect of systemic administrations of ascorbate on the H_2_O_2_ biosensor. 

The current was monitored over a 120-min period as previous investigations have reported this time frame to be suitable for a maximum response to occur [27,42]. This assumption was further validated through implantation of an unmodified 1 mm bare Pt cylinder electrode in the striatum of a NOD SCID mouse. It is obvious from the inset in Figure 5 that the effect of the ascorbate injection reached a maximum within 10–15 min following administration. Furthermore, the current response reached a maximum level ca. 800% from the pre-injection baseline. This effect of ascorbate is absent in the average subtracted H_2_O_2_ biosensor trace illustrated in Figure 5 (*n* = 5). Specifically, the subtracted current was −44 ± 55 pA which corresponded to negligible deviations from pre-injection baseline levels being recorded (see Figure 5B). Overall, there was a 3 ± 3% variation in current that reached a maximum response after 26 ± 17 min and returned to basal levels after 67 ± 23 min. Collectively, these findings support retention of both H_2_O_2_ blank and catalase electrode selectivity in physiological conditions. Moreover, once subtraction was performed, a negligible change in current response was observed supporting the assumption that characteristics reported in vitro have translated across to in vivo conditions recorded in freely moving mice. 

#### 3.4.3. Stability of Baseline H_2_O_2_ Current in the Striatum of Freely Moving NOD SCID Mice

**Figure 6:**
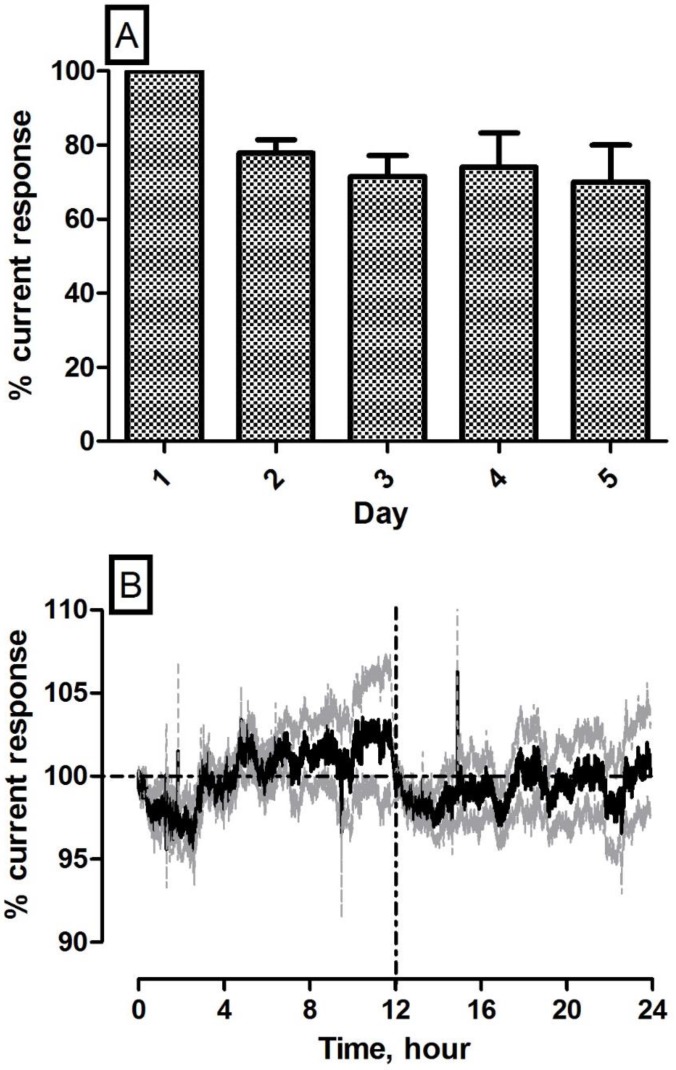
(**A**) Average percentage baseline in vivo data (*n* = 6) for subtracted H_2_O_2_ biosensor recorded in the striatum of NOD SCID mice. Percentage current response data represented as mean ± SEM. (**B**) Averaged 12-h raw data percentage response (lights on (0–12 h), *n* = 14/7), lights off (12–24 h), *n* = 17/7) of subtracted H_2_O_2_ current from biosensors implanted in the striatum of NOD SCID mice. Mean percentage current response represented by black trace, percentage error represented by grey trace.

Efficient stability of the H_2_O_2_ biosensor current over continuous recordings is a prerequisite for eventual deployment in animal models of disease. Previously, we reported the stability of amperometric NO and O_2_ sensor currents over extended periods in the striatum of NOD SCID mice [19]. It is apparent from Figure 6A that a stable baseline trend is obtained from the subtracted H_2_O_2_ current over five days of continuous recording. In summary, Day 1 baselines were normalised to 100% and all subsequent baselines are presented as a percentage of the baseline recorded on Day 1. For the H_2_O_2_ biosensor there was a slight drop in current observed on Day 2 (78 ± 4%) which is in line with ex vivo investigations discussed earlier. A similar phenomenon has been reported previously for in vivo amperometric sensors following initial exposure to physiological conditions [18,19,27,44,45]. Notwithstanding this, no significant variation (*p* = 0.89, one-way ANOVA; 6 animals) was reported over subsequent days (Day 3, 72 ± 6%; Day 4, 74 ± 9%; and Day 5, 70 ± 10%) which demonstrates the stability of the H_2_O_2_ signal over a five-day recording period in NOD SCID mice. These findings corroborate ex vivo studies reported earlier, whereby an initial decrease in H_2_O_2_ detection was observed after 24-h exposure to brain tissue homogenate, however, subsequently there was no significant difference recorded up to seven days exposure for both H_2_O_2_ blank and catalase electrodes. Others have reported on the effect of pre-treating electrochemical surfaces with proteins and lipids prior to implantation in the rodent brain. The principle being to eliminate this initial in vivo sensitivity drop by pre-calibrating the sensors in vitro after a defined period of proteins and lipid exposure [46,47,48]. 

Further analysis identified the average baseline currents for H_2_O_2_ blank electrodes (1871 ± 303 pA, *n* = 6) and H_2_O_2_ catalase electrodes (1104 ± 176 pA, *n* = 6) over the course of the chronic investigations. The subtracted H_2_O_2_ current (767 ± 162, pA, *n* = 6) in freely moving animals corresponds to a baseline H_2_O_2_ concentration of 1.7 ± 0.4 µM and 2.4 ± 0.5 µM based on slope/sensitivity values obtained from in vitro and ex vivo calibration data (seven days exposure) respectively. These concentrations are in good agreement with extracellular concentrations reported by others (3.1 µM) from the dual biosensor implanted in the striatum of freely moving rats [18]. In contrast, findings from the acute investigations identified significantly lower baseline levels present in anaesthetised mice. Specifically, comparable baseline levels were recorded for H_2_O_2_ blank electrodes ((256 ± 25 pA, *n* = 4) and (462 ± 36 pA, *n* = 5)) and H_2_O_2_ catalase electrodes ((470 ± 43 pA, *n* = 4) and (414 ± 47 pA, *n* = 5)) over the course of acute perfusions and infusions respectively. These resulted in subtracted H_2_O_2_ currents that support a theory that basal levels of H_2_O_2_ are extremely low throughout anaesthesia induction. This is most probably attributed to decreased H_2_O_2_ availability as a result of reduced neuronal activity [19,49] and mitochondrial function [50,51,52] which are two principal sources of production [2]. Hitherto, the question of functional H_2_O_2_ concentrations in the intra and extracellular compartments of brain tissue have not been resolved, however, evidence exists to support an intracellular concentration range of 15 to 150 µM without inducing oxidative damage [2]. How this translates to extracellular levels is unknown at present but our findings suggest significantly lower levels of H_2_O_2_ diffuse across into the ECF. 

#### 3.4.4. Twenty-Four-Hour Recording of H_2_O_2_ Current Dynamics in the Striatum of Freely Moving NOD SCID Mice

Continuous 24-h amperometric recordings were examined to investigate H_2_O_2_ current dynamics over extended periods of inactivity (light cycle) and increased locomotor activity (night cycle). We previously reported on diurnal variations in NOD SCID mice using amperometric NO and O_2_ sensors, a phenomenon we attributed to increased neuronal activation and local cerebral blood flow during nocturnal periods. It is apparent from the pooled data recorded during the light phase (*n* = 14/7) and dark phase (*n* = 17/7) that the amperometric current deviates little from baseline levels over the course of the 24-h period (see Figure 6A). These findings suggest the absence of diurnal modulations in H_2_O_2_ current in NOD SCID mouse striatum which may be indicative of a highly efficient and intricate antioxidant mechanism that functions meticulously to control basal levels of ROS. Antioxidant enzymes are critical for protection against oxidative stress induced by ROS and like a myriad of neurochemical species, their mechanisms of action and concentrations may fluctuate during light and dark phases. 

A number of groups have investigated the effect of circadian rhythms on antioxidants [11,33,53,54] but none have directly investigated the effect of the 24-h cycle on ROS levels. All have reported variations in the numerous antioxidant defence systems across a 24-h period which may be representative of the dynamics associated with scavenging oxidant molecules such as H_2_O_2_. In particular, Baydas et al. reported increased glutathione peroxidase activity in rat brain during the dark phase to perhaps compensate for increased ROS production during this period [33]. Similarly, Sani and colleagues illustrated two peak times for catalase activity during a 24-h monitoring period which could be indicative of increased scavenging of H_2_O_2_ produced at different intervals throughout the day/night cycle [11]. In parallel, the neurohormone melatonin plays a critical role in regulating circadian rhythms leading to the tight control of endogenous antioxidant mechanisms. Constant exposure of animals to light has been observed to abolish the night cycle rise in melatonin and thus leads to a reduction in the night time enzymatic activity of glutathione peroxidase and superoxide dismutase [55]. Our 24-h current recordings tentatively support these assumptions that H_2_O_2_ production is tightly controlled by a highly efficient antioxidant mechanism at different stages over a 24-h period. Further work is necessary to determine the exact mechanisms responsible, however, these initial findings highlight the potential ability of the H_2_O_2_ biosensor to measure long term neurochemical dynamics in freely moving NOD SCID mice. 

## 4. Conclusions

The principle objective of the work described within was to validate the performance of a previously characterised amperometric H_2_O_2_ biosensor in the striatum of immunocompromised NOD SCID mice. In vitro investigations demonstrated high sensitivity for H_2_O_2_ and selectivity against AA at physiologically relevant concentrations. Ex vivo investigations supported reliable recordings for up to seven days in brain tissue for both H_2_O_2_ blank and catalase electrodes in relation to H_2_O_2_ detection and AA rejection characteristics. Furthermore, the physical effect of implanting the catalase modified electrode into mouse brain tissue did not compromise the immobilisation of the enzyme on the electrode surface. Initial in vivo characterisation studies were performed in anaesthetised animals to circumvent concerns around the toxicity associated with exogenously administered H_2_O_2_ and inhibitors of antioxidant enzymes. Microdialysis and microinfusion probes were combined with the amperometric H_2_O_2_ biosensor to locally deliver the target analyte and inhibitor compounds to the mouse striatum. The combined microinfusion/H_2_O_2_ biosensor construct provided far superior findings and confirmed the in vivo detection of increasing H_2_O_2_ concentrations at the H_2_O_2_ biosensor surface. Subsequently, the performance of the H_2_O_2_ biosensor was characterised in the striatum of freely moving NOD SCID mice. A transient deviation from baseline was reported for both control saline and sodium ascorbate interference injections. The latter confirms the integrity of the permselective rejection layer was retained once the H_2_O_2_ biosensor was implanted which is critical for the in vivo characterisation of the design. The stability of the H_2_O_2_ biosensor current was confirmed over a five-day period, which corroborates our ex vivo findings. Furthermore, analysis of 24-h signal recordings identified the absence of diurnal variations for the biosensor. Nevertheless, the latter supports the potential ability of the amperometric biosensor to measure long-term neurochemical variations in immunocompromised mice.

## Figures and Tables

**Table 1:** Summary of in vivo characterisation data for H_2_O_2_ biosensor co-implanted with microdialysis probe in the striatum of anaesthetised NOD SCID mice.

[H_2_O_2_]	∆*I* (pA)	% ↑ H_2_O_2_ Current	% H_2_O_2_ Current vs. Baseline	% H_2_O_2_ Current vs. aCSF
aCSF	−73 ± 80	−2 ± 2	*p* = 0.49	-
100 µM	45 ± 40	6 ± 2	* *p* < 0.05	* *p* < 0.05
500 µM	131 ± 74	9 ± 2	* *p* < 0.05	** *p* < 0.01
1 mM	90 ± 34	9 ± 2	* *p* < 0.05	** *p* < 0.01
10 mM	47 ± 8	22 ± 8	*p* = 0.06	* *p* < 0.05
1 mM MSA	18 ± 2	11 ± 3	*p* = 0.09	* *p* < 0.05

* Denotes level of significance.

**Table 2:** Summary of in vivo characterisation data for H_2_O_2_ biosensor co-implanted with microinfusion probe in the striatum of anaesthetised NOD SCID mice.

(H_2_O_2_)	∆*I* (nA)	% ↑ H_2_O_2_ Current	% H_2_O_2_ Current vs. Baseline	% H_2_O_2_ Current vs. aCSF
aCSF	−5 ± 24 (pA)	−7 ± 4	*p* = 0.18	-
20 µM	0.56 ± 0.25	184 ± 56	* *p* < 0.05	** *p* < 0.01
50 µM	0.82 ± 0.44	359 ± 107	* *p* < 0.05	** *p* < 0.01
100 µM	1.94 ± 0.68	1941 ± 675	*p* = 0.06	* *p* < 0.05
1 mM	27.22 ± 8.30	9262 ± 2814	* *p* < 0.05	* *p* < 0.05
10 mM	139.95 ± 28.94	39507 ± 7365	** *p* < 0.01	*** *p* < 0.001
1 mM SA	10.96 ± 6.19	2439 ± 558	* *p* < 0.05	*** *p* < 0.001
1 mM MSA	6.75 ± 3.10	1782 ± 422	*p* = 0.05	** *p* < 0.01

* Denotes level of significance.

## Supplementary Materials

The following are available online at www.mdpi.com/1424-8220/17/7/1596/s1.
