# Supplementary material for: Real-Time Amperometric Recording of Extracellular H2O2 in the Brain of Immunocompromised Mice: An In Vitro, Ex Vivo and In Vivo Characterisation Study
Source: Sensors (Basel). 2017 Jul 8;17(7):1596. doi: 10.3390/s17071596 (PMC5539478; doi:10.3390/s17071596)
Supplement: Supplementary file 1 [file sensors-17-01596-s001.pdf]

Supplementary Materials: Real-Time Amperometric Recording of Extracellular H_2O_2 in the Brain of Immunocompromised Mice: An In Vitro, Ex Vivo and In Vivo Characterisation Study

Caroline H. Reid and Niall J. Finnerty *

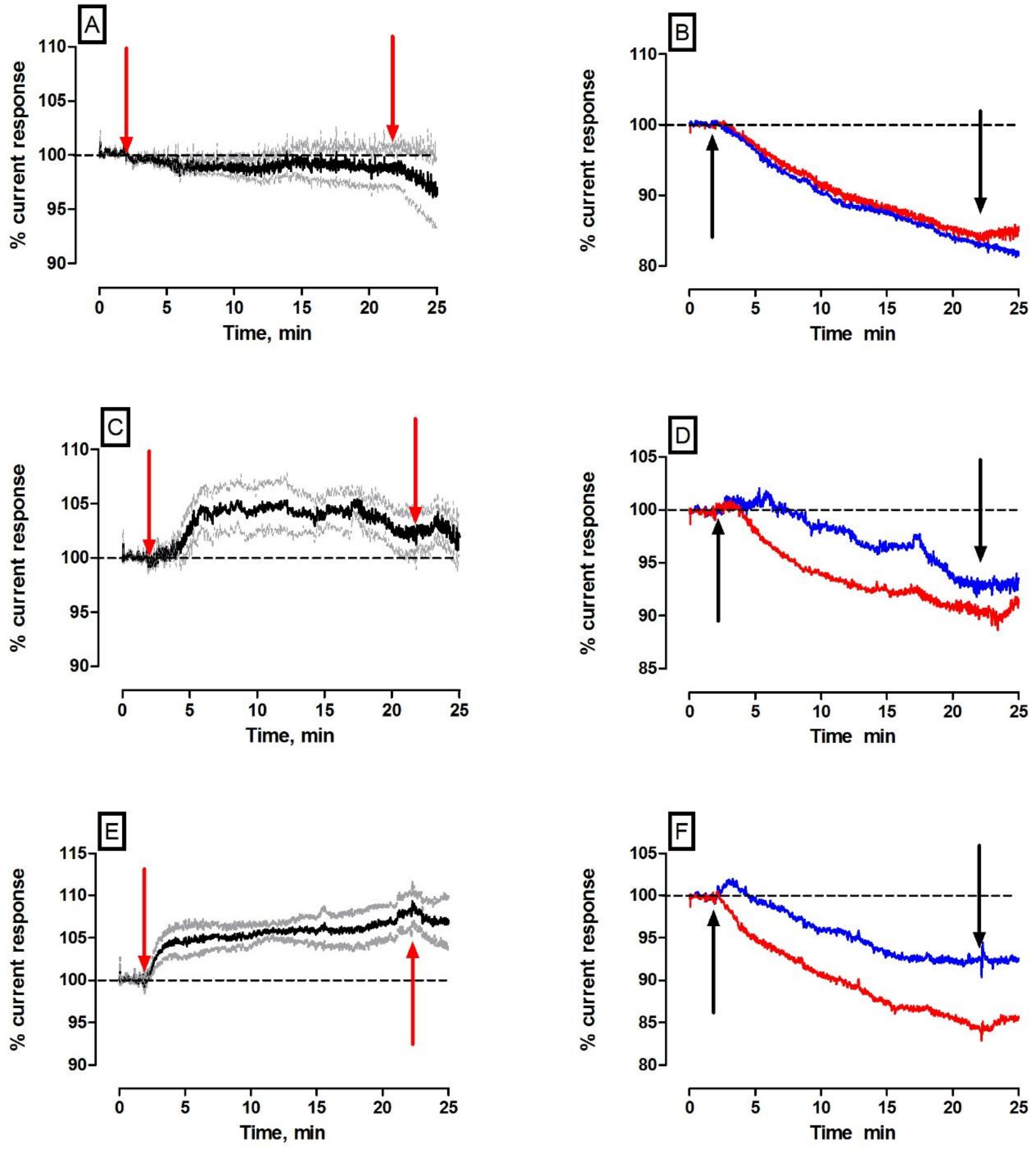

Figure S1. Averaged raw data % current response of H_2O_2 sensors co-implanted with microdialysis probe following 20 min aCSF perfusion (**A** – subtracted H_2O_2 trace, **B** – H_2O_2 blank electrode (blue trace) & catalase electrode (red trace)), 20 min 100 μM H_2O_2/aCSF perfusion (**C** – subtracted H_2O_2 trace, **D** – H_2O_2 blank electrode (blue trace) & catalase electrode (red trace)) and 20 min 500 μM H_2O_2/aCSF perfusion (**E** – subtracted H_2O_2 trace, **F** – H_2O_2 blank electrode (blue trace) & catalase electrode (red trace)) in the striatum of anaesthetised NOD SCID mice (*n* = 4). Arrows indicate start/end of perfusion. **A**, **C**, **E** – mean % current response represented by black trace, % error represented by grey trace. **B**, **D**, **F** – mean % current responses-for clarity % errors have been excluded from these traces.

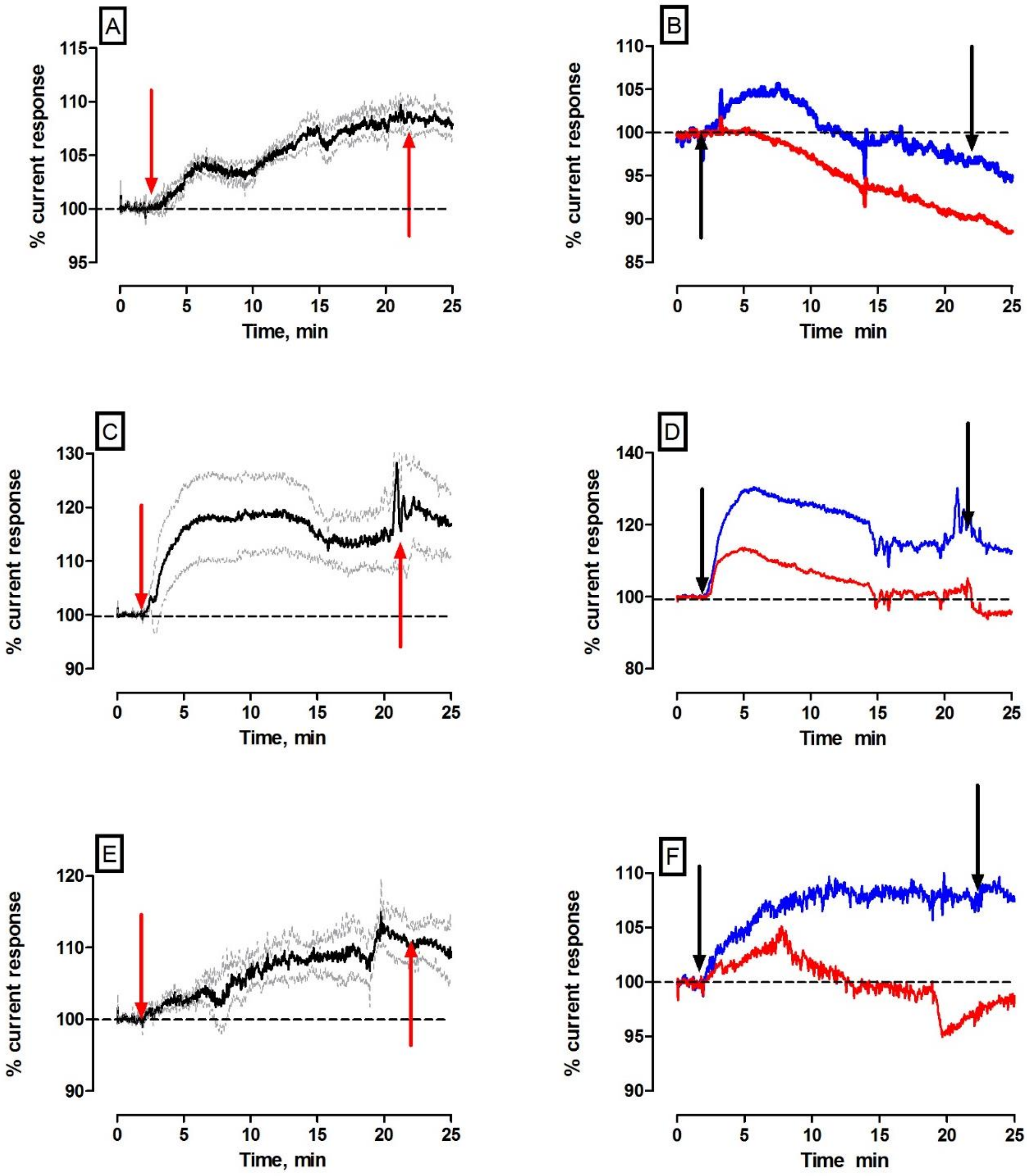

Figure S2. Averaged raw data % current response of H_2O_2 sensors co-implanted with microdialysis probe following 20 min 1 mM H_2O_2/aCSF perfusion (**A** – subtracted H_2O_2 trace, **B** – H_2O_2 blank electrode (blue trace) & catalase electrode (red trace)), 20 min 10 mM H_2O_2/aCSF perfusion (**C** – subtracted H_2O_2 trace, **D** – H_2O_2 blank electrode (blue trace) & catalase electrode (red trace)) (n = 4) and 20 min 1 mM MSA/aCSF perfusion (left – subtracted H_2O_2 trace, right – H_2O_2 blank electrode (blue trace) & catalase electrode (red trace)) (n = 3). Arrows indicate start/end of perfusion. **A**, **C**, **E** – mean % current response represented by black trace, % error represented by grey trace. **B**, **D**, **F** – mean % current responses-for clarity % errors have been excluded from these traces.

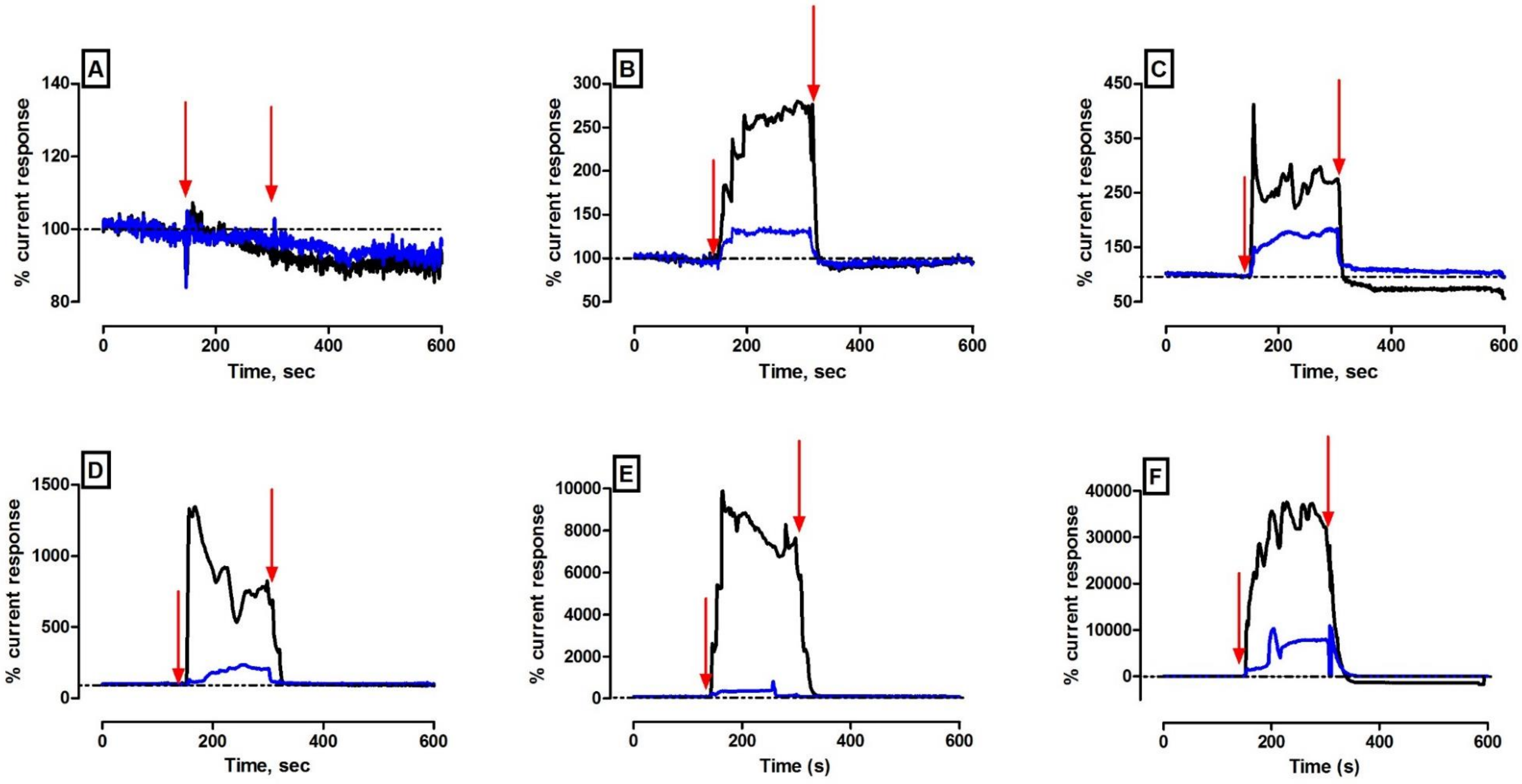

Figure S3. Averaged raw data % current response of H_2O_2 sensors co-implanted with microinfusion probe following 150 s aCSF perfusion (A – H_2O_2 blank electrode (black trace) & catalase electrode (blue trace)), 150 s 20 μM H_2O_2/aCSF perfusion (B – H_2O_2 blank electrode (black trace) & catalase electrode (blue trace)), 150 s 50 μM H_2O_2/aCSF perfusion (C – H_2O_2 blank electrode (black trace) & catalase electrode (blue trace)), 150 s 100 μM H_2O_2/aCSF perfusion (D – H_2O_2 blank electrode (black trace) & catalase electrode (blue trace)), 150 s 1 mM H_2O_2/aCSF perfusion (E – H_2O_2 blank electrode (black trace) & catalase electrode (blue trace)), 150 s 10 mM H_2O_2/aCSF perfusion (F – H_2O_2 blank electrode (black trace) & catalase electrode (blue trace)) in the striatum of anaesthetised NOD SCID mice (n = 4). Arrows indicate start/end of infusion. Data presented as mean % current response-for clarity % errors have been excluded from these traces.

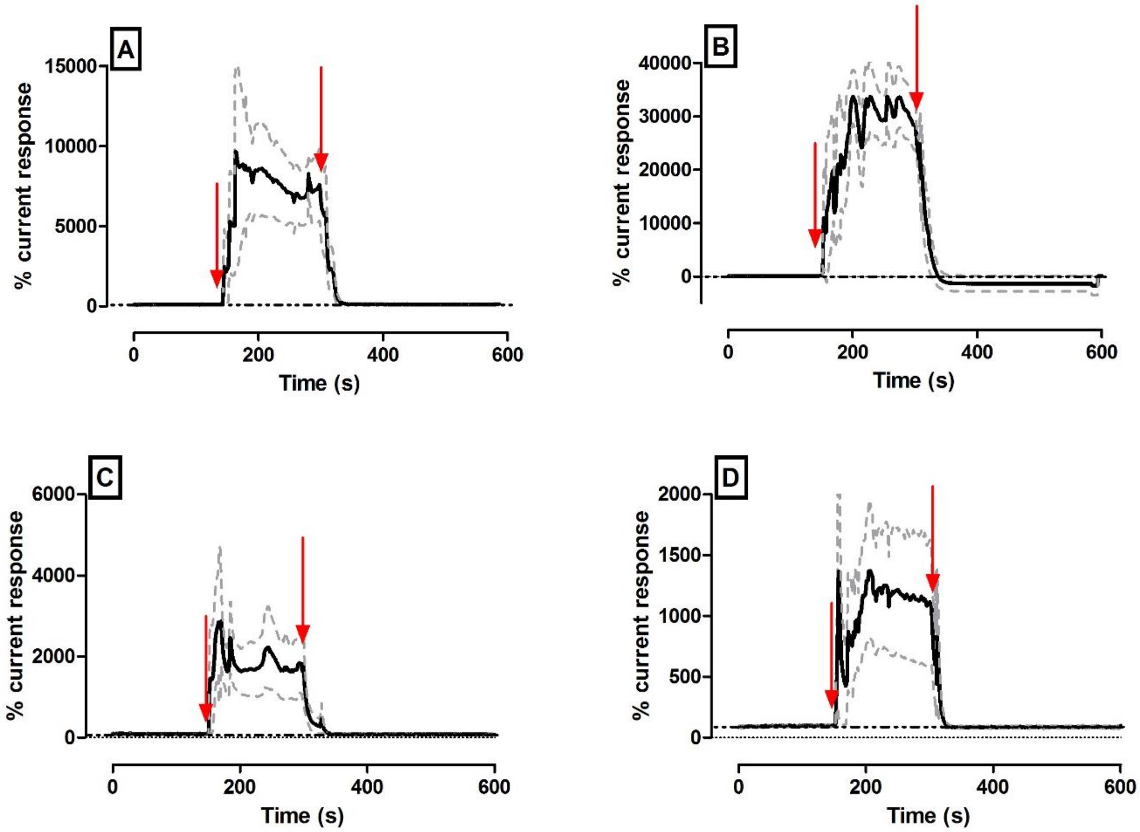

Figure S4. Averaged raw data % current response of subtracted H_2O_2 biosensor co-implanted with microinfusion probe following 150 s infusion of (**A**) 1 mM H_2O_2/aCSF (**B**) 10 mM H_2O_2/aCSF (**C**) 1 mM SA and (**D**) 1 mM MSA. Arrows indicate start/end of infusion. Mean % current response represented by black trace, % error represented by grey trace.

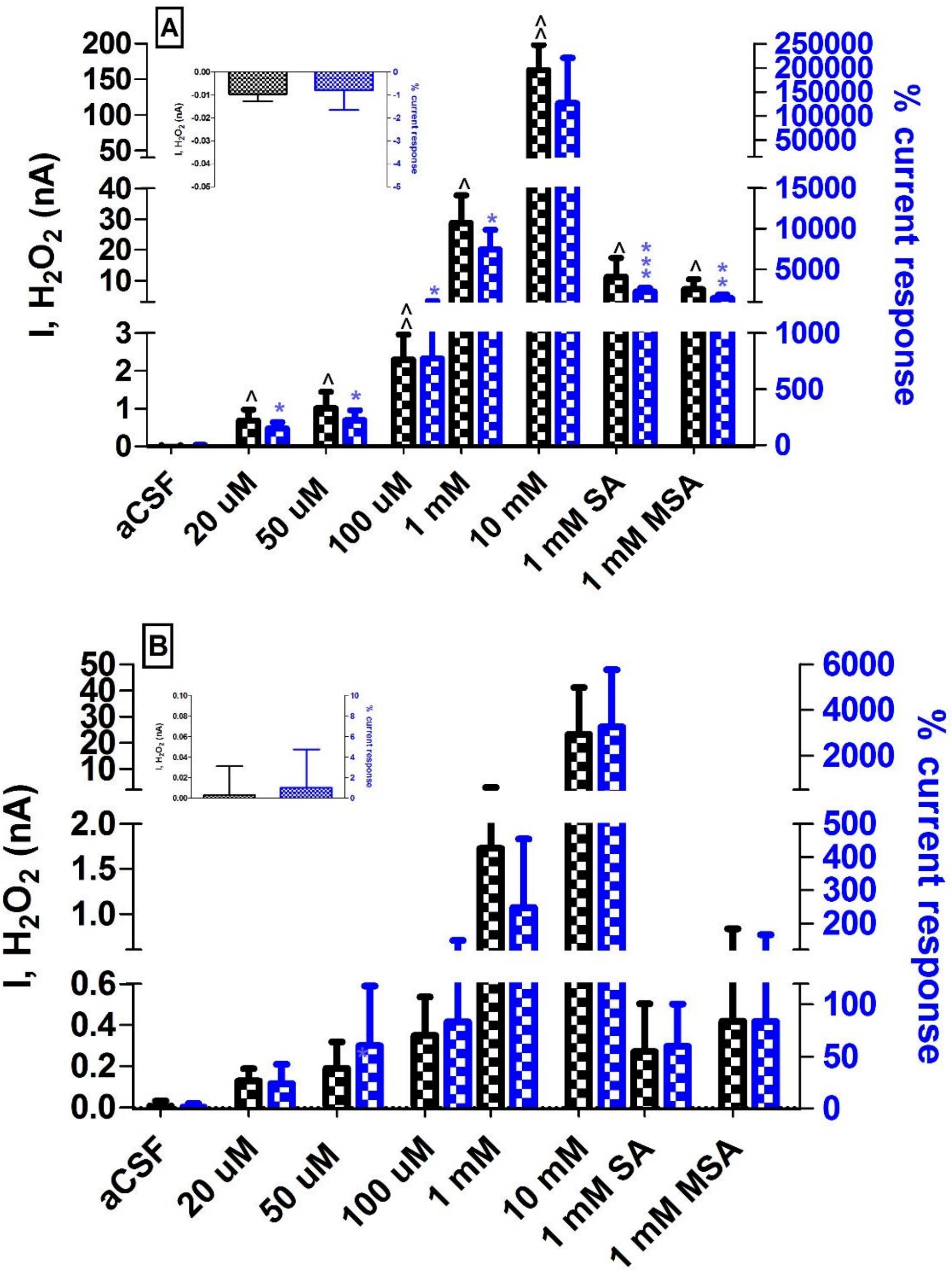

Figure S5. Dose effect of infusion of 0–10 mM H_2O_2 / aCSF (*n* = 5), 1 mM SA (*n* = 3) and MSA (*n* = 3) on (**A**) H_2O_2 blank electrode current and (**B**) H_2O_2 catalase electrode current. Current (left y-axis) and % current response (right *y*-axis) data represented as mean ± SEM. ^ and * denote level of significance for currents and % currents respectively.

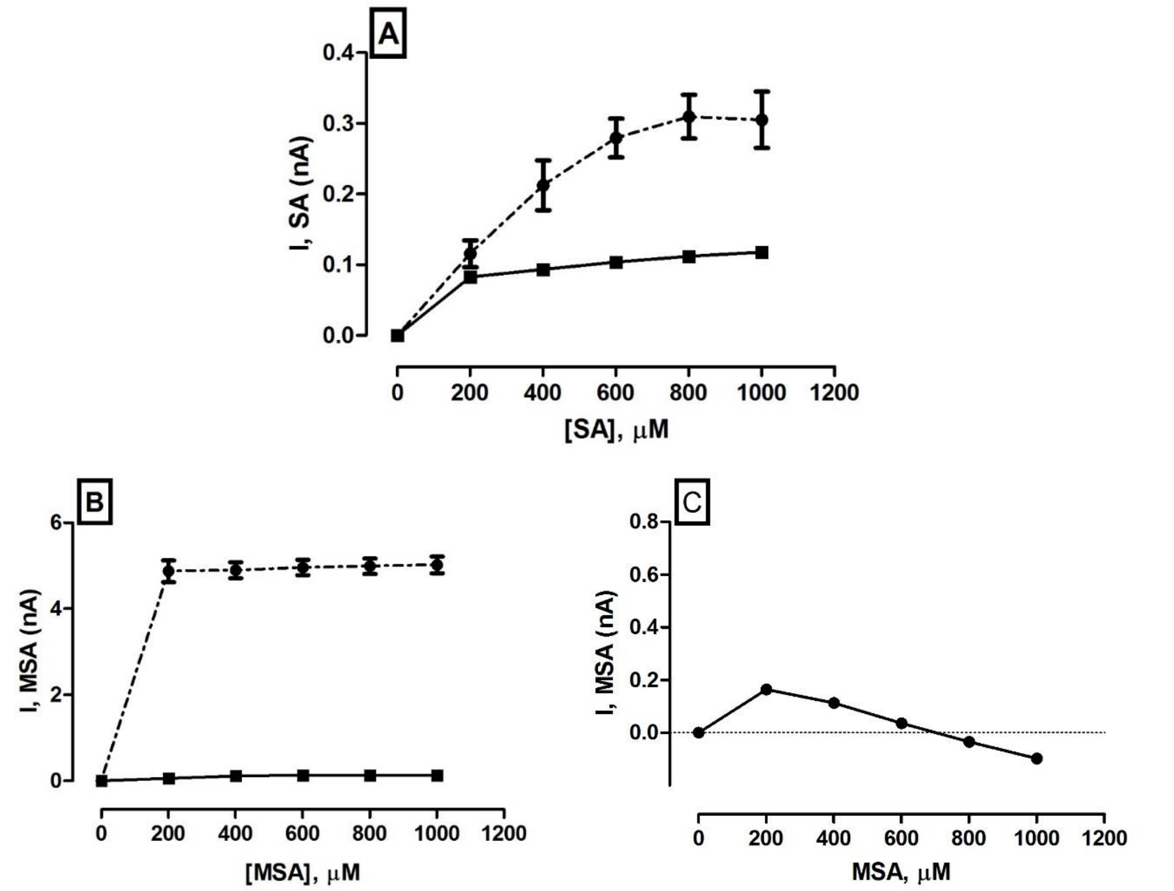

Figure S6. Current concentration profiles for (**A**) 0–1000 μM SA calibration and (**B**) 0–1000 μM MSA calibrations on unmodified bare Pt electrodes (dashed lines, *n* = 8) and H_2O_2 blank electrodes (solid lines, *n* = 8). (**C**) Effect of 0–10 mM H_2O_2 calibration on MSA rejection characteristics of H_2O_2 blank electrodes (*n* = 12). All concentration profile data is presented as mean ± SEM.
